# Supplementary material for: Evolutionary Study of Protein Short Tandem Repeats in Protein Families
Source: Biomolecules. 2023 Jul 13;13(7):1116. doi: 10.3390/biom13071116 (PMC10377733; doi:10.3390/biom13071116)
Supplement: Supplementary file 1 [file biomolecules-13-01116-s001.zip › Suppl.FileS1.zip!/Suppl.File1/OG11/OG11_results.pdf]

All epSTR

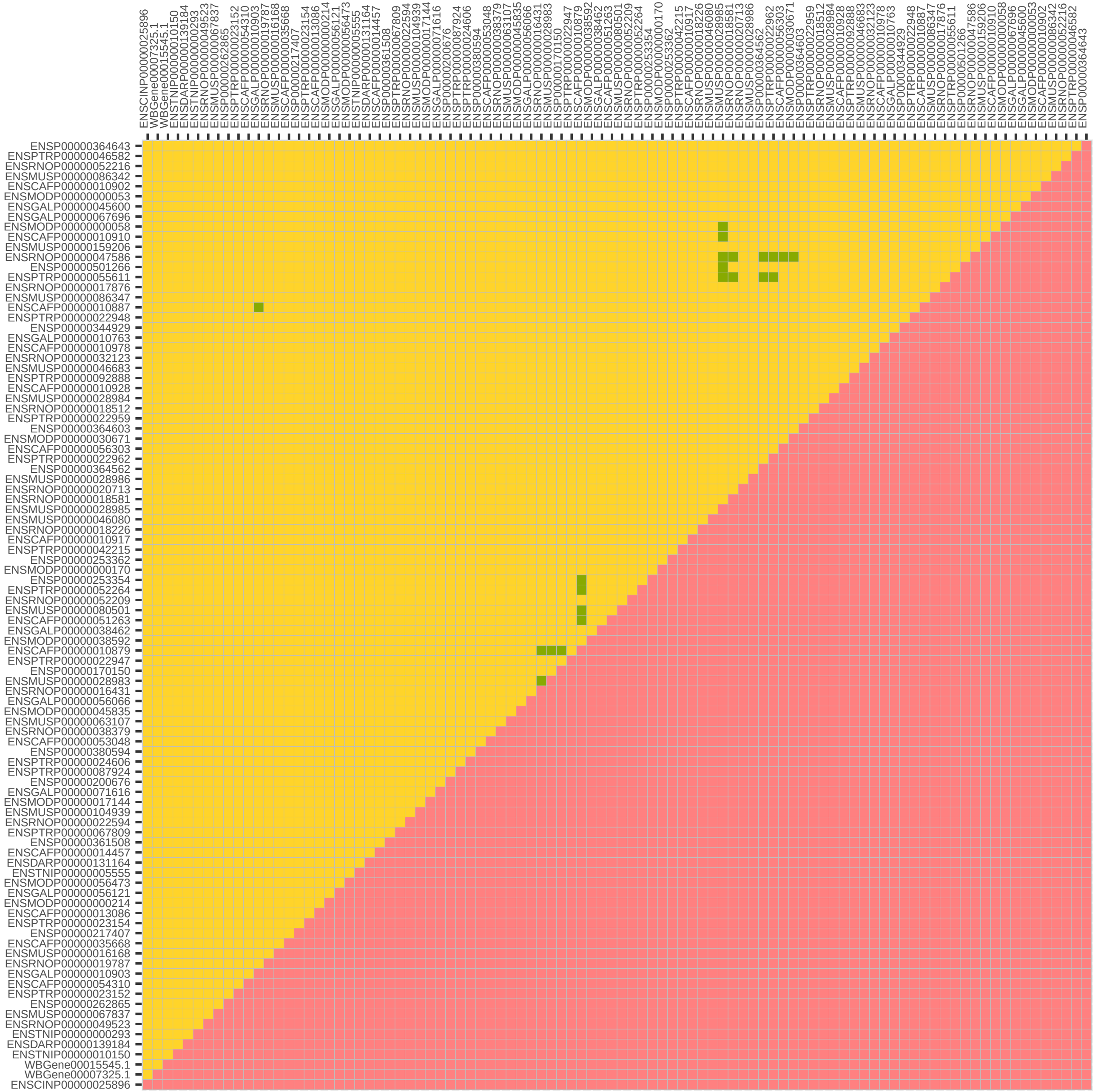

ENSCINP00000025896
WBGene00007325.1
WBGene00015545.1
ENSTNIP00000010150
ENSDARP00000139184
ENSTNIP00000000293
ENSRNOP00000049523
ENSMUSP00000067837
ENSP00000262865
ENSPTRP00000023152
ENSCAFP00000054310
ENSGALP00000010903
ENSRNOP00000019787
ENSMUSP00000016168
ENSCAFP00000035668
ENSP00000217407
ENSPTRP00000023154
ENSCAFP00000013086
ENSMODP00000000214
ENSGALP00000056121
ENSMODP00000056473
ENSTNIP00000005555
ENSDARP00000131164
ENSCAFP00000014457
ENSP00000361508
ENSPTRP00000067809
ENSRNOP00000022594
ENSMUSP00000104939
ENSMODP00000017144
ENSGALP00000071616
ENSP00000200676
ENSPTRP00000087924
ENSPTRP00000024606
ENSP00000380594
ENSCAFP00000053048
ENSRNOP00000038379
ENSMUSP00000063107
ENSMODP00000045835
ENSGALP00000056066
ENSRNOP00000016431
ENSMUSP00000028983
ENSP00000170150
ENSPTRP00000022947
ENSCAFP00000010879
ENSMODP00000038592
ENSGALP00000038462
ENSCAFP00000051263
ENSMUSP00000080501
ENSRNOP00000052209
ENSPTRP00000052264
ENSP00000253354
ENSMODP00000000170
ENSP00000253362
ENSPTRP00000042215
ENSCAFP00000010917
ENSRNOP00000018226
ENSMUSP00000046080
ENSMUSP00000028985
ENSRNOP00000018581
ENSRNOP00000020713
ENSMUSP00000028986
ENSP00000364562
ENSPTRP00000022962
ENSCAFP00000056303
ENSMODP00000030671
ENSP00000364603
ENSPTRP00000022959
ENSRNOP00000018512
ENSMUSP00000028984
ENSCAFP00000010928
ENSPTRP00000092888
ENSMUSP00000046683
ENSRNOP00000032123
ENSCAFP00000010978
ENSGALP00000010763
ENSP00000344929
ENSPTRP00000022948
ENSCAFP00000010887
ENSMUSP00000086347
ENSRNOP00000017876
ENSPTRP00000055611
ENSP00000501266
ENSRNOP00000047586
ENSMUSP00000159206
ENSCAFP00000010910
ENSMODP00000000058
ENSGALP00000067696
ENSGALP00000045600
ENSMODP00000000053
ENSCAFP00000010902
ENSMUSP00000086342
ENSRNOP00000052216
ENSPTRP00000046582
ENSP00000364643
