# Supplementary material for: Evolutionary Study of Protein Short Tandem Repeats in Protein Families
Source: Biomolecules. 2023 Jul 13;13(7):1116. doi: 10.3390/biom13071116 (PMC10377733; doi:10.3390/biom13071116)
Supplement: Supplementary file 1 [file biomolecules-13-01116-s001.zip › Suppl.FileS1.zip!/Suppl.File1/OG12/OG12_results.pdf]

All epSTR

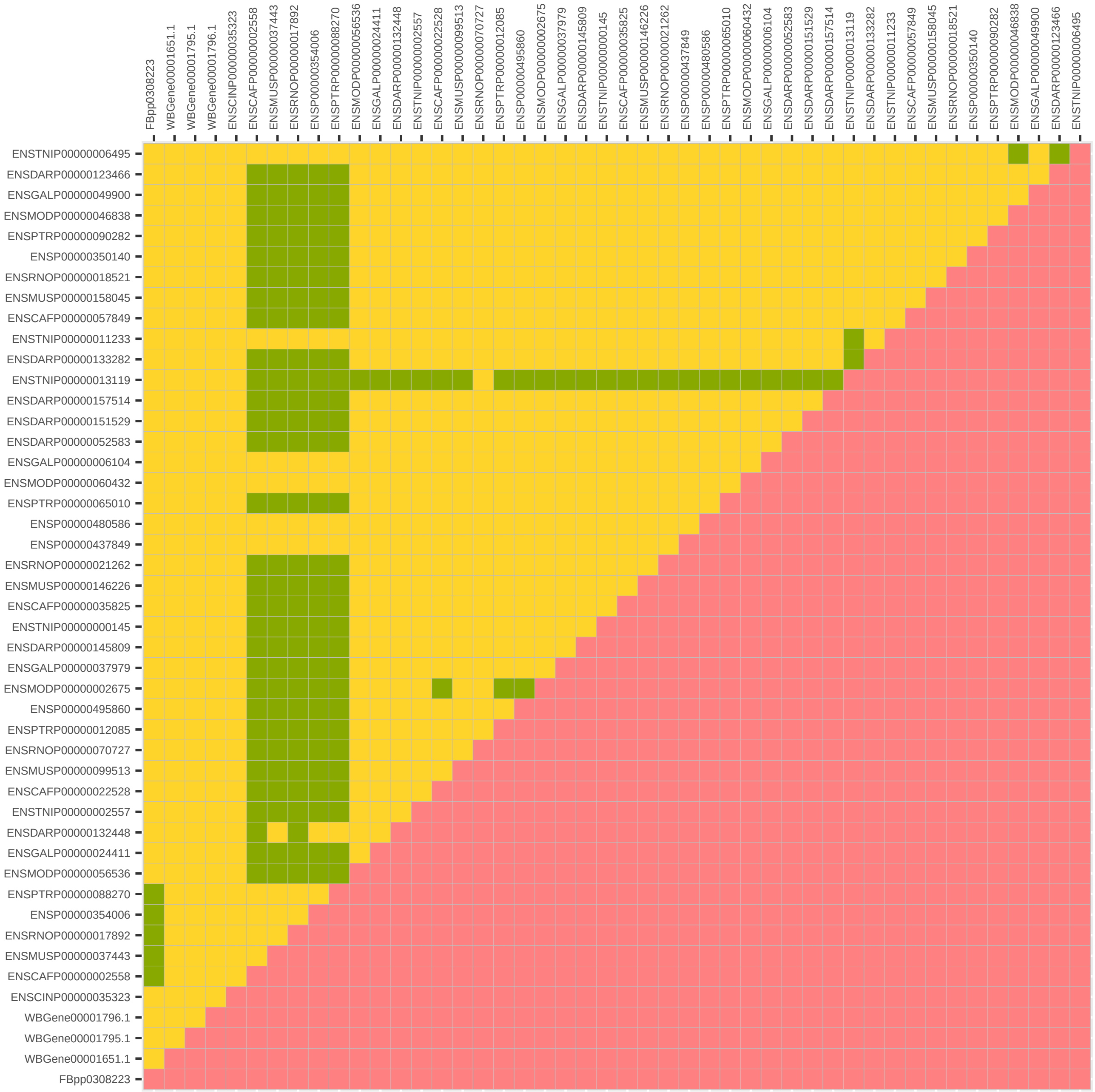
FBpp0308223
WBGene00001651.1
WBGene00001795.1
WBGene00001796.1
ENSCINP00000035323
ENSCAFP00000002558
ENSMUSP00000037443
ENSRNOP00000017892
ENSP00000354006
ENSPTRP00000088270
ENSMODP00000056536
ENSGALP00000024411
ENSDARP00000132448
ENSTNIP00000002557
ENSCAFP00000022528
ENSMUSP00000099513
ENSRNOP00000070727
ENSPTRP00000012085
ENSP00000495860
ENSMODP00000002675
ENSGALP00000037979
ENSDARP00000145809
ENSTNIP00000000145
ENSCAFP00000035825
ENSMUSP00000146226
ENSRNOP00000021262
ENSP00000437849
ENSP00000480586
ENSPTRP00000065010
ENSMODP00000060432
ENSGALP00000006104
ENSDARP00000052583
ENSDARP00000151529
ENSDARP00000157514
ENSTNIP00000013119
ENSDARP00000133282
ENSTNIP00000011233
ENSCAFP00000057849
ENSMUSP00000158045
ENSRNOP00000018521
ENSP00000350140
ENSPTRP00000090282
ENSMODP00000046838
ENSGALP00000049900
ENSDARP00000123466
ENSTNIP00000006495
