# Supplementary material for: Evolutionary Study of Protein Short Tandem Repeats in Protein Families
Source: Biomolecules. 2023 Jul 13;13(7):1116. doi: 10.3390/biom13071116 (PMC10377733; doi:10.3390/biom13071116)
Supplement: Supplementary file 1 [file biomolecules-13-01116-s001.zip › Suppl.FileS1.zip!/Suppl.File1/OG13/OG13_results.pdf]

All epSTR

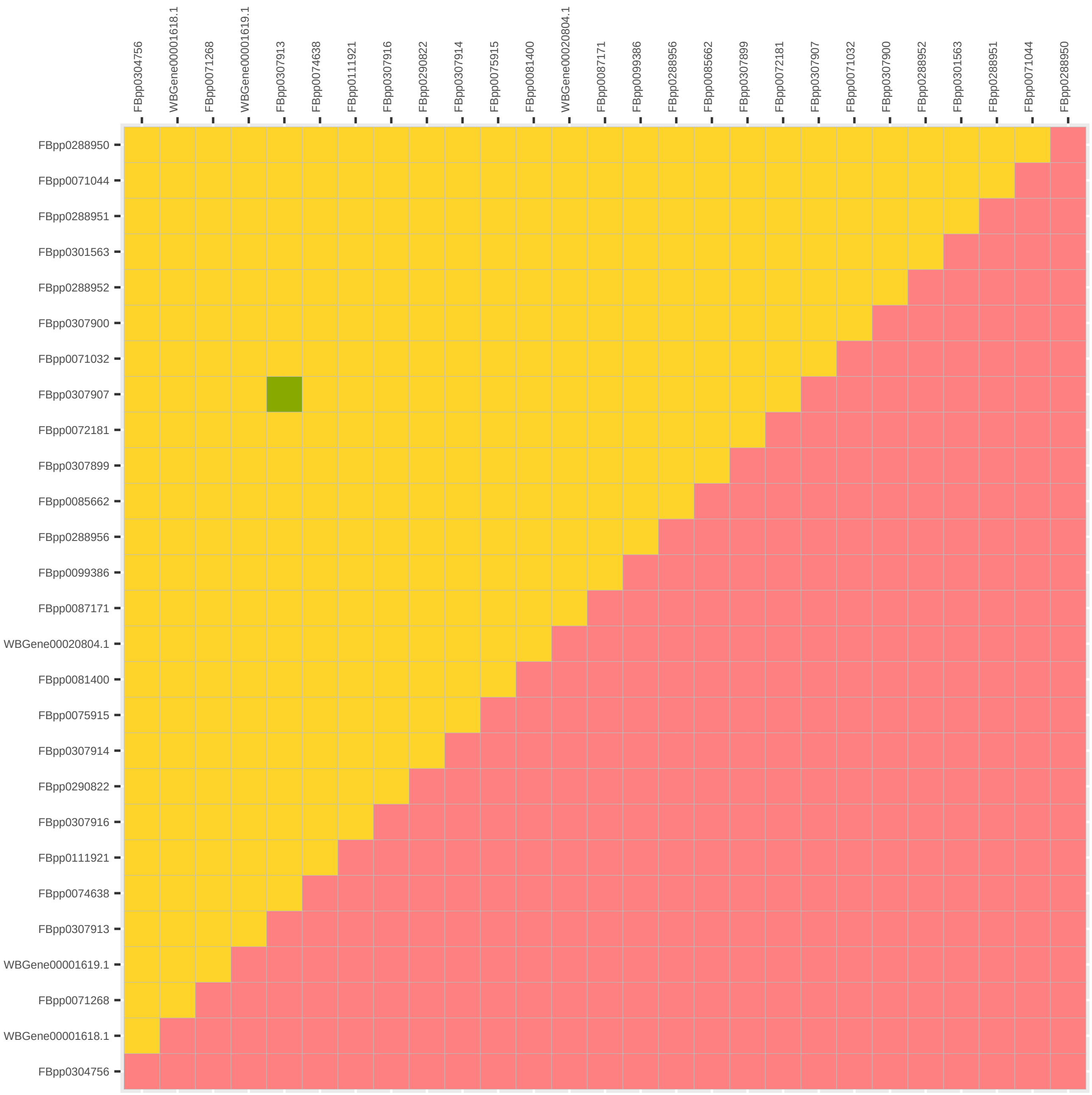

FBpp0304756
WBGene00001618.1
FBpp0071268
WBGene00001619.1
FBpp0307913
FBpp0074638
FBpp0111921
FBpp0307916
FBpp0290822
FBpp0307914
FBpp0075915
FBpp0081400
WBGene00020804.1
FBpp0087171
FBpp0099386
FBpp0288956
FBpp0085662
FBpp0307899
FBpp0072181
FBpp0307907
FBpp0071032
FBpp0307900
FBpp0288952
FBpp0301563
FBpp0288951
FBpp0071044
FBpp0288950
