# Supplementary material for: Evolutionary Study of Protein Short Tandem Repeats in Protein Families
Source: Biomolecules. 2023 Jul 13;13(7):1116. doi: 10.3390/biom13071116 (PMC10377733; doi:10.3390/biom13071116)
Supplement: Supplementary file 1 [file biomolecules-13-01116-s001.zip › Suppl.FileS1.zip!/Suppl.File1/OG14/OG14_results.pdf]

All epSTR

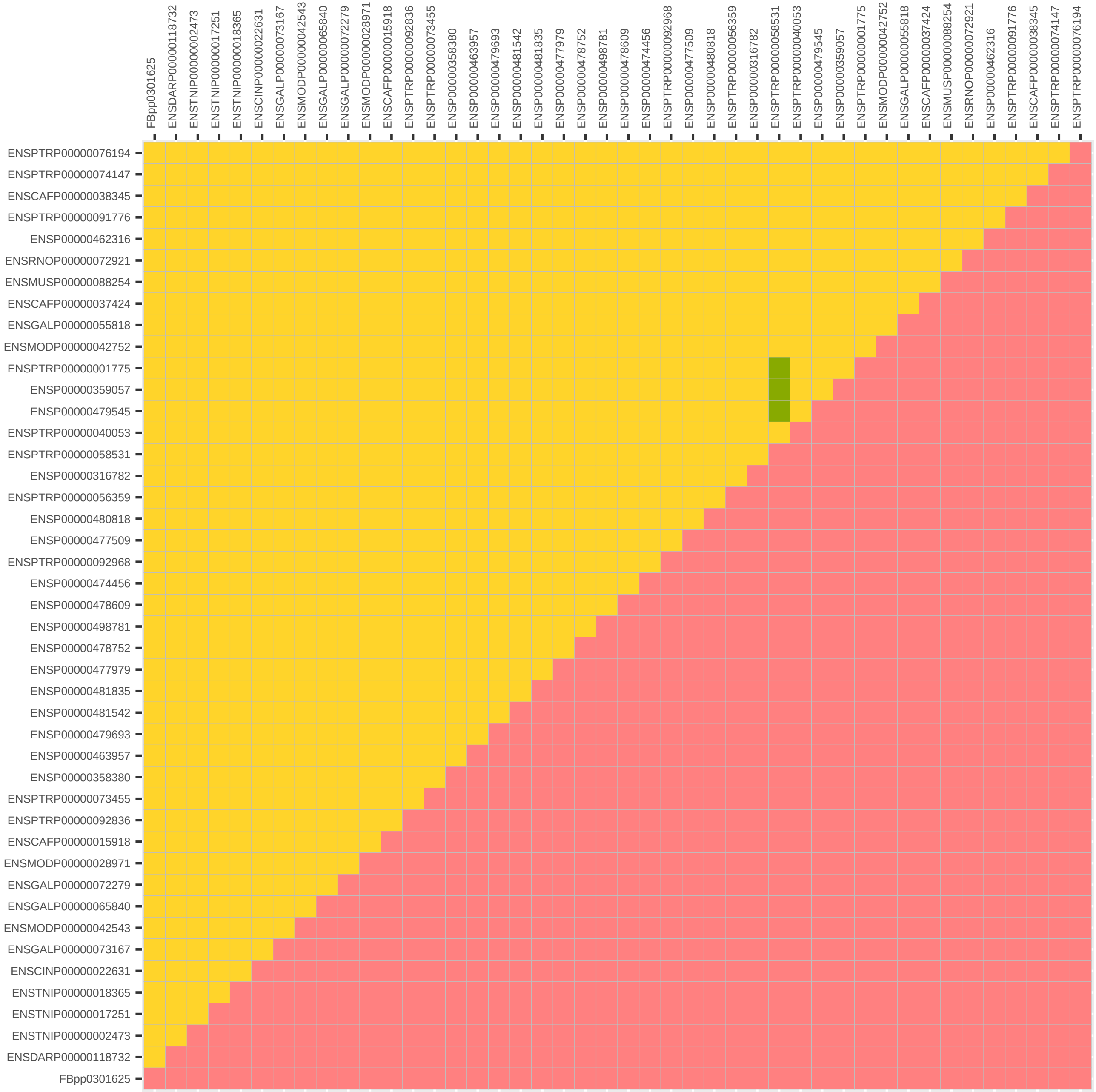
FBpp0301625
ENSDARP00000118732
ENSTNIP00000002473
ENSTNIP00000017251
ENSTNIP00000018365
ENSCINP00000022631
ENSGALP00000073167
ENSMODP00000042543
ENSGALP00000065840
ENSGALP00000072279
ENSMODP00000028971
ENSCAFP00000015918
ENSPTRP00000092836
ENSPTRP00000073455
ENSP00000358380
ENSP00000463957
ENSP00000479693
ENSP00000481542
ENSP00000481835
ENSP00000477979
ENSP00000478752
ENSP00000498781
ENSP00000478609
ENSP00000474456
ENSPTRP00000092968
ENSP00000477509
ENSP00000480818
ENSPTRP00000056359
ENSP00000316782
ENSPTRP00000058531
ENSPTRP00000040053
ENSP00000479545
ENSP00000359057
ENSPTRP00000001775
ENSMODP00000042752
ENSGALP00000055818
ENSCAFP00000037424
ENSMUSP00000088254
ENSRNOP00000072921
ENSP00000462316
ENSPTRP00000091776
ENSCAFP00000038345
ENSPTRP00000074147
ENSPTRP00000076194
