# Supplementary material for: Evolutionary Study of Protein Short Tandem Repeats in Protein Families
Source: Biomolecules. 2023 Jul 13;13(7):1116. doi: 10.3390/biom13071116 (PMC10377733; doi:10.3390/biom13071116)
Supplement: Supplementary file 1 [file biomolecules-13-01116-s001.zip › Suppl.FileS1.zip!/Suppl.File1/OG15/OG15_results.pdf]

All epSTR

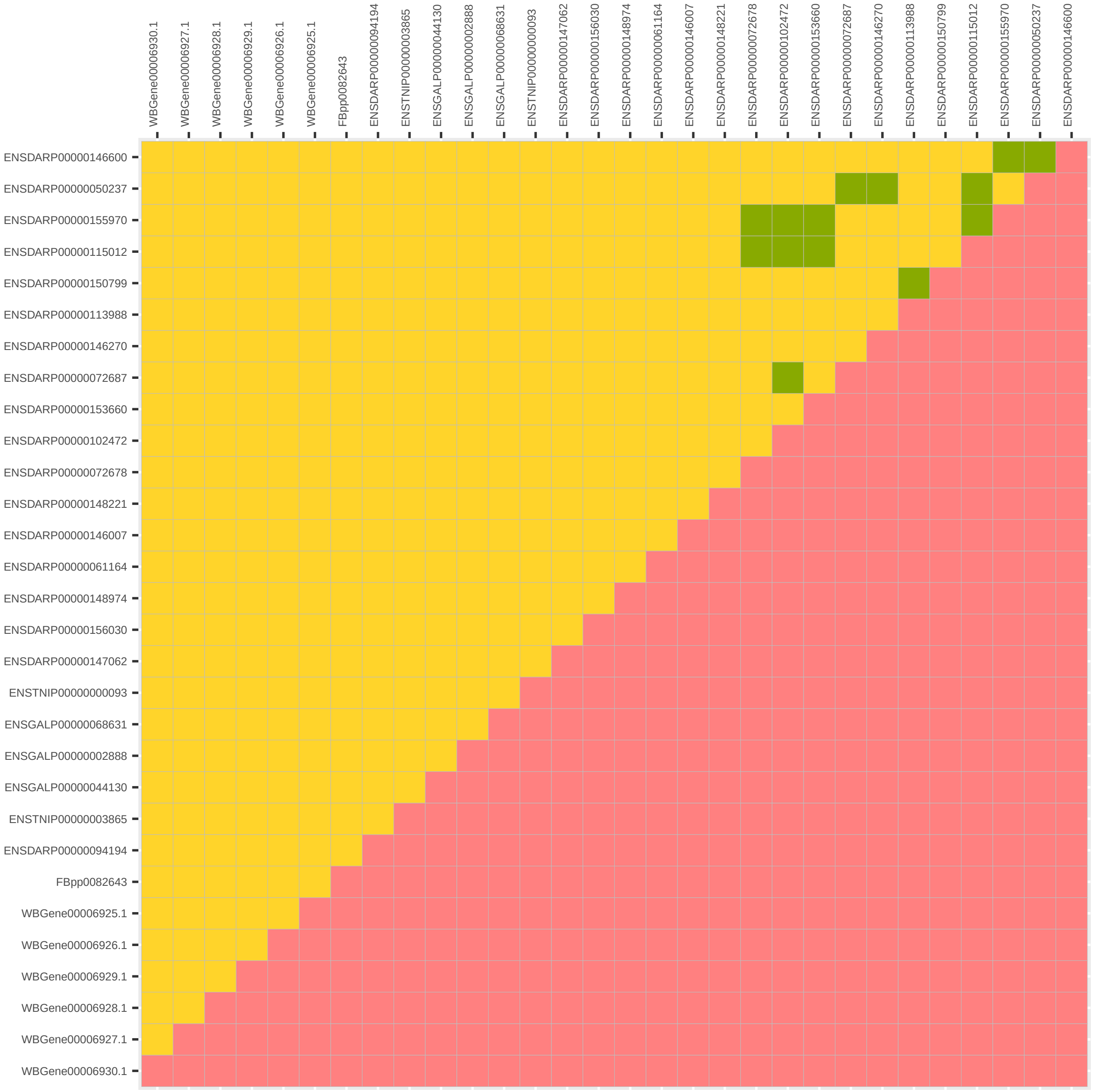

WBGene00006930.1
WBGene00006927.1
WBGene00006928.1
WBGene00006929.1
WBGene00006926.1
WBGene00006925.1
FBpp0082643
ENSDARP00000094194
ENSTNIP00000003865
ENSGALP00000044130
ENSGALP00000002888
ENSGALP00000068631
ENSTNIP00000000093
ENSDARP00000147062
ENSDARP00000156030
ENSDARP00000148974
ENSDARP00000061164
ENSDARP00000146007
ENSDARP00000148221
ENSDARP00000072678
ENSDARP00000102472
ENSDARP00000153660
ENSDARP00000072687
ENSDARP00000146270
ENSDARP00000113988
ENSDARP00000150799
ENSDARP00000115012
ENSDARP00000155970
ENSDARP00000050237
ENSDARP00000146600
