# Supplementary material for: Evolutionary Study of Protein Short Tandem Repeats in Protein Families
Source: Biomolecules. 2023 Jul 13;13(7):1116. doi: 10.3390/biom13071116 (PMC10377733; doi:10.3390/biom13071116)
Supplement: Supplementary file 1 [file biomolecules-13-01116-s001.zip › Suppl.FileS1.zip!/Suppl.File1/OG17/OG17_results.pdf]

All epSTR

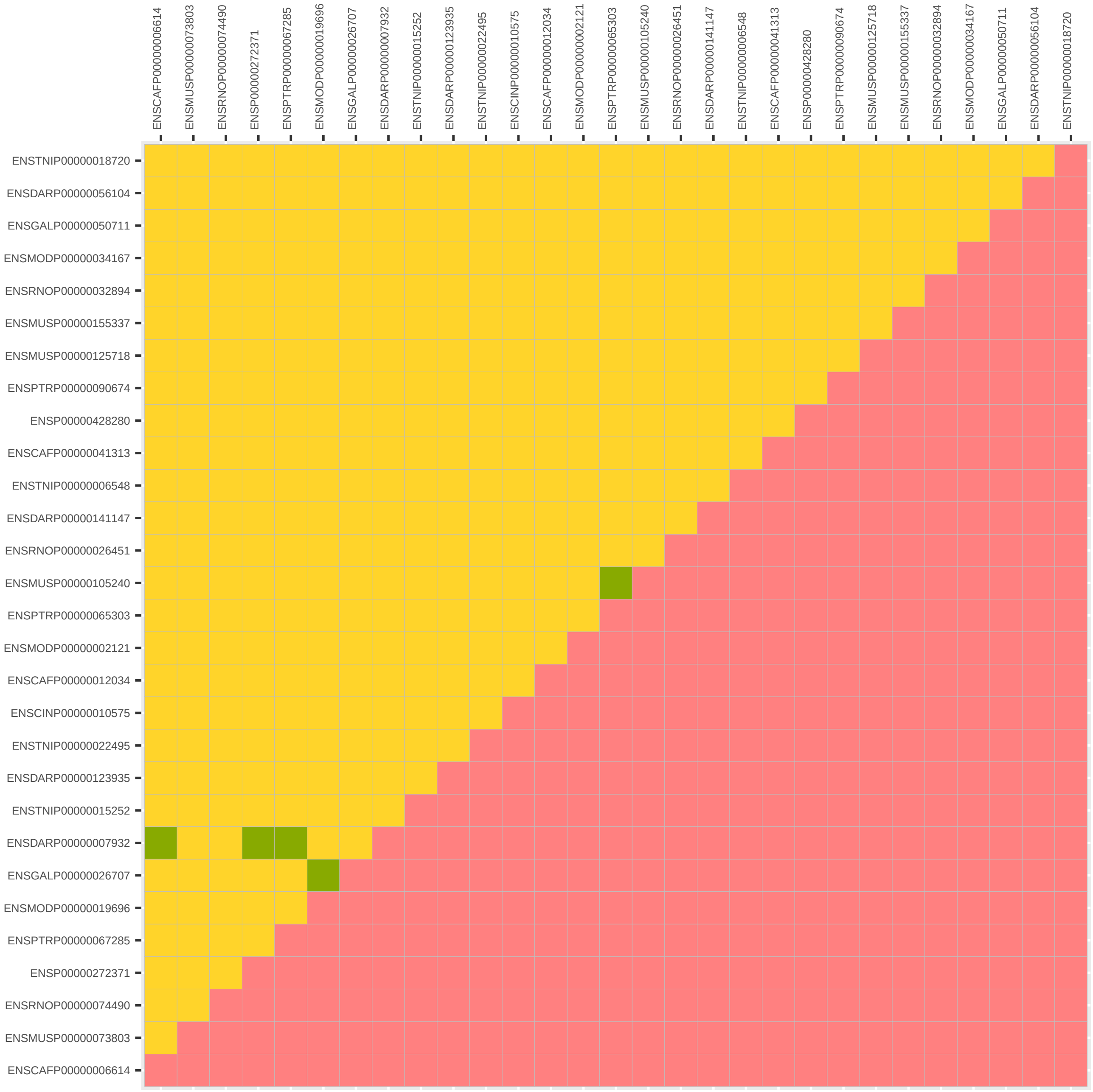

ENSCAFP00000006614
ENSMUSP00000073803
ENSRNOP00000074490
ENSP00000272371
ENSPTRP00000067285
ENSMODP00000019696
ENSGALP00000026707
ENSDARP00000007932
ENSTNIP00000015252
ENSDARP00000123935
ENSTNIP00000022495
ENSCINP00000010575
ENSCAFP00000012034
ENSMODP00000002121
ENSPTRP00000065303
ENSMUSP00000105240
ENSRNOP00000026451
ENSDARP00000141147
ENSTNIP00000006548
ENSCAFP00000041313
ENSP00000428280
ENSPTRP00000090674
ENSMUSP00000125718
ENSMUSP00000155337
ENSRNOP00000032894
ENSMODP00000034167
ENSGALP00000050711
ENSDARP00000056104
ENSTNIP00000018720
