# Supplementary material for: Evolutionary Study of Protein Short Tandem Repeats in Protein Families
Source: Biomolecules. 2023 Jul 13;13(7):1116. doi: 10.3390/biom13071116 (PMC10377733; doi:10.3390/biom13071116)
Supplement: Supplementary file 1 [file biomolecules-13-01116-s001.zip › Suppl.FileS1.zip!/Suppl.File1/OG20/OG20_results.pdf]

All epSTR

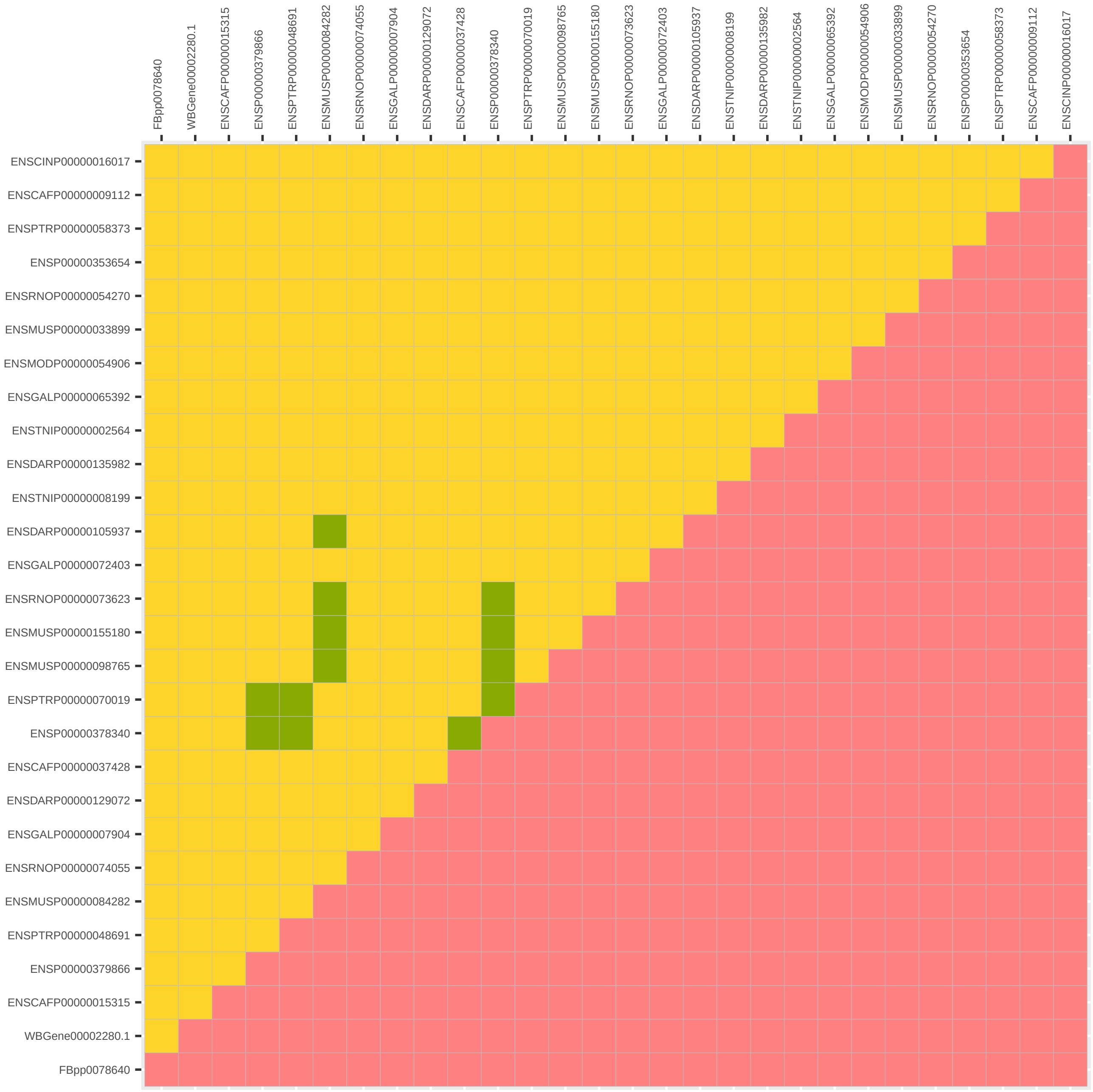

FBpp0078640
WBGene00002280.1
ENSCAFP00000015315
ENSP00000379866
ENSPTRP00000048691
ENSMUSP00000084282
ENSRNOP00000074055
ENSGALP00000007904
ENSDARP00000129072
ENSCAFP00000037428
ENSP00000378340
ENSPTRP00000070019
ENSMUSP00000098765
ENSMUSP00000155180
ENSRNOP00000073623
ENSGALP00000072403
ENSDARP00000105937
ENSTNIP00000008199
ENSDARP00000135982
ENSTNIP00000002564
ENSGALP00000065392
ENSMODP00000054906
ENSMUSP00000033899
ENSRNOP00000054270
ENSP00000353654
ENSPTRP00000058373
ENSCAFP00000009112
ENSCINP00000016017
