# Supplementary material for: Evolutionary Study of Protein Short Tandem Repeats in Protein Families
Source: Biomolecules. 2023 Jul 13;13(7):1116. doi: 10.3390/biom13071116 (PMC10377733; doi:10.3390/biom13071116)
Supplement: Supplementary file 1 [file biomolecules-13-01116-s001.zip › Suppl.FileS1.zip!/Suppl.File1/OG22/OG22_results.pdf]

All epSTR

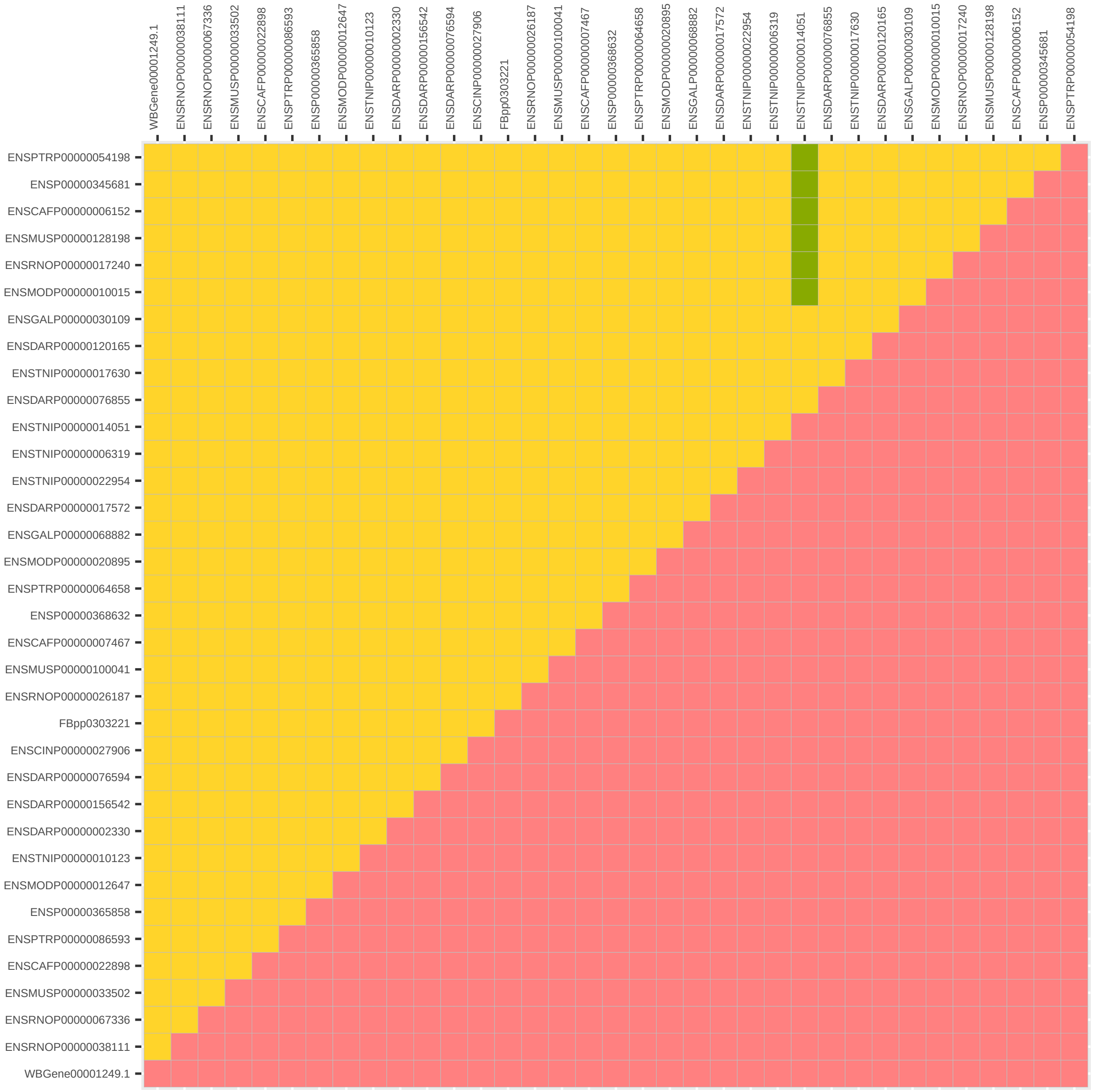
WBGene00001249.1
ENSRNOP00000038111
ENSRNOP00000067336
ENSMUSP00000033502
ENSCAFP00000022898
ENSPTRP00000086593
ENSP00000365858
ENSMODP00000012647
ENSTNIP00000010123
ENSDARP00000002330
ENSDARP00000156542
ENSDARP00000076594
ENSCINP00000027906
FBpp0303221
ENSRNOP00000026187
ENSMUSP00000100041
ENSCAFP00000007467
ENSP00000368632
ENSPTRP00000064658
ENSMODP00000020895
ENSGALP00000068882
ENSDARP00000017572
ENSTNIP00000022954
ENSTNIP00000006319
ENSTNIP00000014051
ENSDARP00000076855
ENSTNIP00000017630
ENSDARP00000120165
ENSGALP00000030109
ENSMODP00000010015
ENSRNOP00000017240
ENSMUSP00000128198
ENSCAFP00000006152
ENSP00000345681
ENSPTRP00000054198
