# Supplementary material for: Evolutionary Study of Protein Short Tandem Repeats in Protein Families
Source: Biomolecules. 2023 Jul 13;13(7):1116. doi: 10.3390/biom13071116 (PMC10377733; doi:10.3390/biom13071116)
Supplement: Supplementary file 1 [file biomolecules-13-01116-s001.zip › Suppl.FileS1.zip!/Suppl.File1/OG23/OG23_results.pdf]

All epSTR

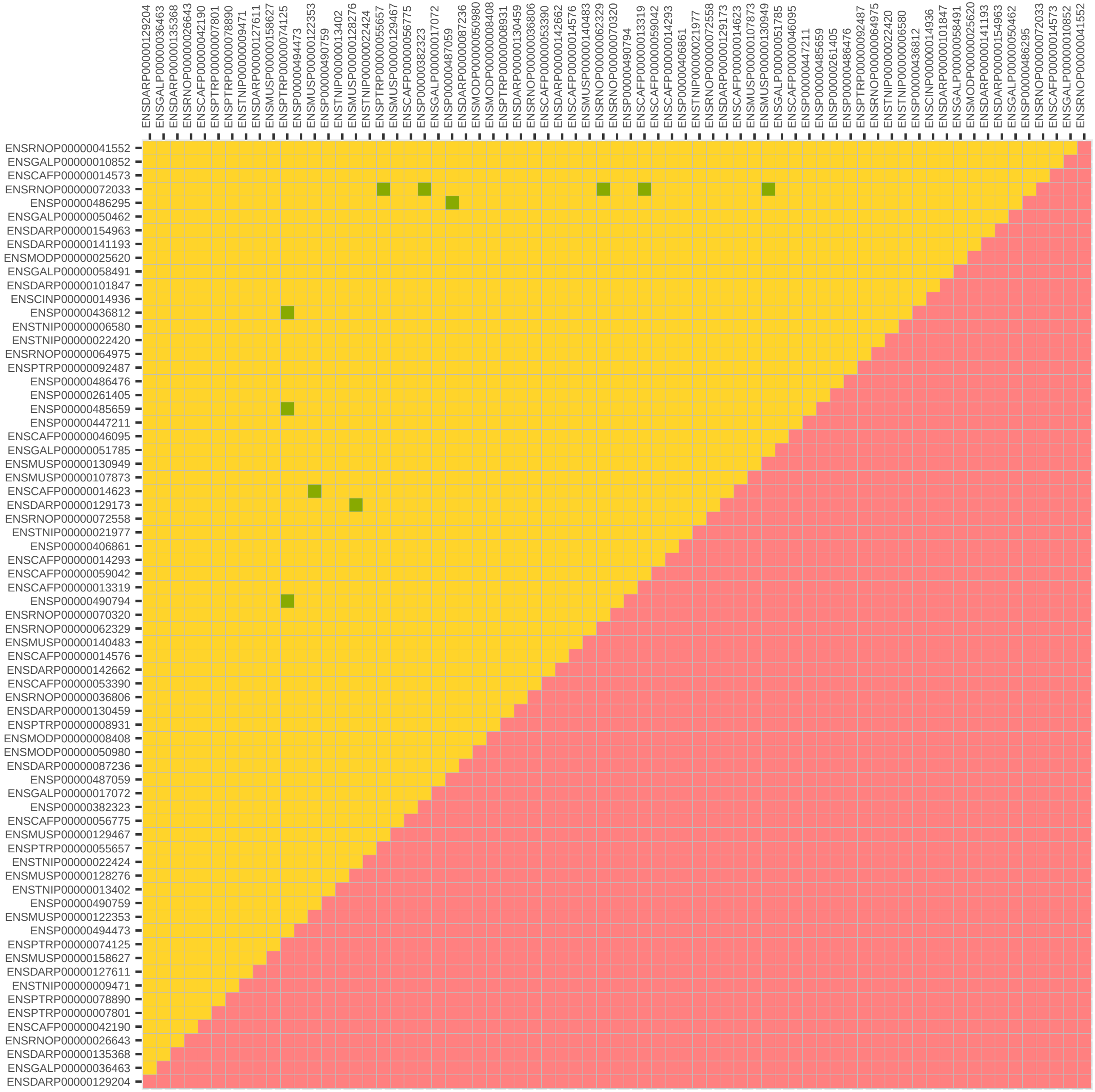
ENSDARP00000129204
ENSGALP00000036463
ENSDARP00000135368
ENSRNOP00000026643
ENSCAFP00000042190
ENSPTRP00000007801
ENSPTRP00000078890
ENSTNIP00000009471
ENSDARP00000127611
ENSMUSP00000158627
ENSPTRP00000074125
ENSP00000494473
ENSMUSP00000122353
ENSP00000490759
ENSTNIP00000013402
ENSMUSP00000128276
ENSTNIP00000022424
ENSPTRP00000055657
ENSMUSP00000129467
ENSCAFP00000056775
ENSP00000382323
ENSGALP00000017072
ENSP00000487059
ENSDARP00000087236
ENSMODP00000050980
ENSMODP00000008408
ENSPTRP00000008931
ENSDARP00000130459
ENSRNOP00000036806
ENSCAFP00000053390
ENSDARP00000142662
ENSCAFP00000014576
ENSMUSP00000140483
ENSRNOP00000062329
ENSRNOP00000070320
ENSP00000490794
ENSCAFP00000013319
ENSCAFP00000059042
ENSCAFP00000014293
ENSP00000406861
ENSTNIP00000021977
ENSRNOP00000072558
ENSDARP00000129173
ENSCAFP00000014623
ENSMUSP00000107873
ENSMUSP00000130949
ENSGALP00000051785
ENSCAFP00000046095
ENSP00000447211
ENSP00000485659
ENSP00000261405
ENSP00000486476
ENSPTRP00000092487
ENSRNOP00000064975
ENSTNIP00000022420
ENSTNIP00000006580
ENSP00000436812
ENSCINP00000014936
ENSDARP00000101847
ENSGALP00000058491
ENSMODP00000025620
ENSDARP00000141193
ENSDARP00000154963
ENSGALP00000050462
ENSP00000486295
ENSRNOP00000072033
ENSCAFP00000014573
ENSGALP00000010852
ENSRNOP00000041552
