# Supplementary material for: Evolutionary Study of Protein Short Tandem Repeats in Protein Families
Source: Biomolecules. 2023 Jul 13;13(7):1116. doi: 10.3390/biom13071116 (PMC10377733; doi:10.3390/biom13071116)
Supplement: Supplementary file 1 [file biomolecules-13-01116-s001.zip › Suppl.FileS1.zip!/Suppl.File1/OG36/OG36_results.pdf]

All epSTR

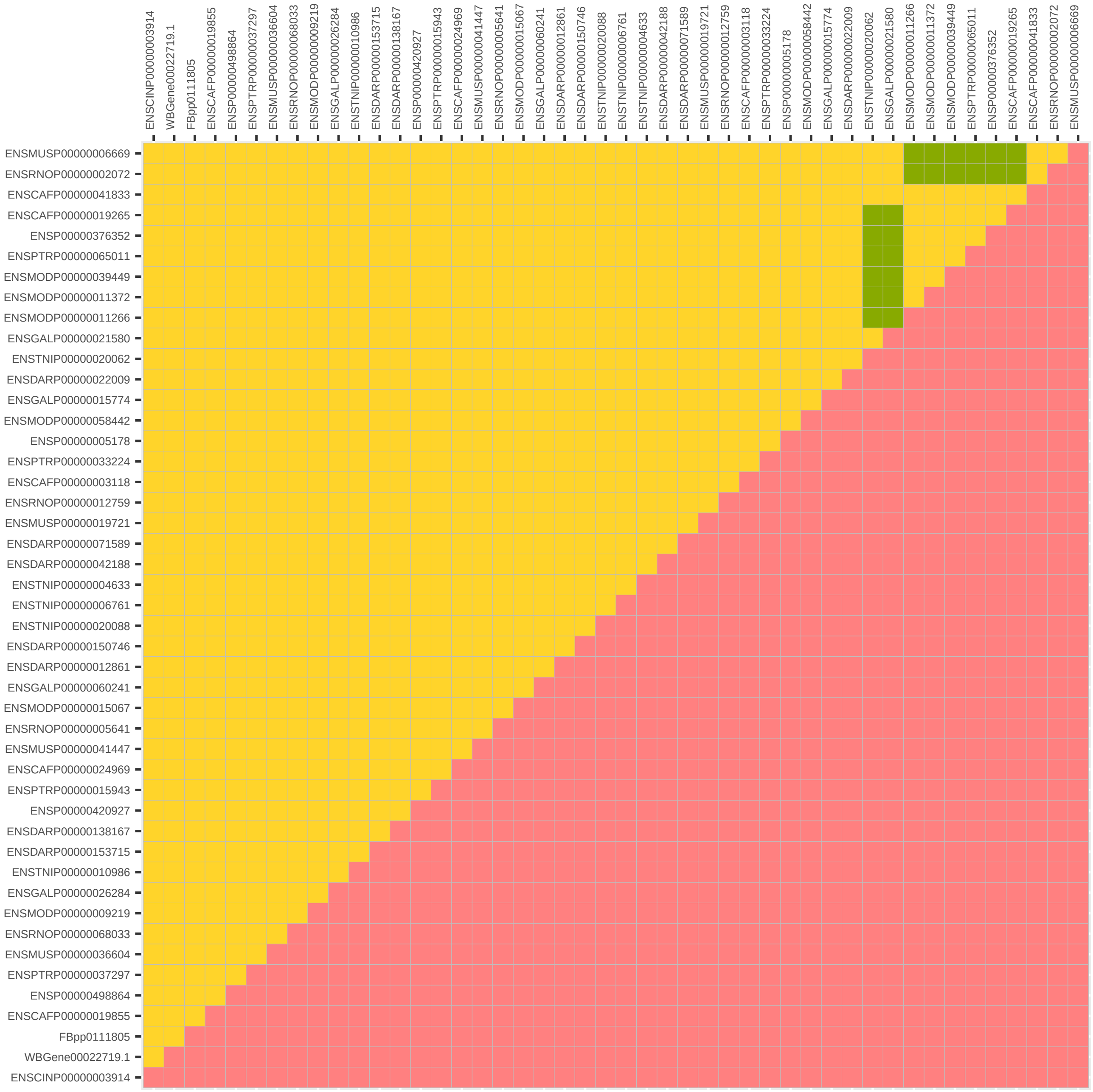
ENSCINP00000003914
WBGene00022719.1
FBpp0111805
ENSCAFP00000019855
ENSP00000498864
ENSPTRP00000037297
ENSMUSP00000036604
ENSRNOP00000068033
ENSMODP00000009219
ENSGALP00000026284
ENSTNIP00000010986
ENSDARP00000153715
ENSDARP00000138167
ENSP00000420927
ENSPTRP00000015943
ENSCAFP00000024969
ENSMUSP00000041447
ENSRNOP00000005641
ENSMODP00000015067
ENSGALP00000060241
ENSDARP00000012861
ENSDARP00000150746
ENSTNIP00000020088
ENSTNIP00000006761
ENSTNIP00000004633
ENSDARP00000042188
ENSDARP00000071589
ENSMUSP00000019721
ENSRNOP00000012759
ENSCAFP00000003118
ENSPTRP00000033224
ENSP00000005178
ENSMODP00000058442
ENSGALP00000015774
ENSDARP00000022009
ENSTNIP00000020062
ENSGALP00000021580
ENSMODP00000011266
ENSMODP00000011372
ENSMODP00000039449
ENSPTRP00000065011
ENSP00000376352
ENSCAFP00000019265
ENSCAFP00000041833
ENSRNOP00000002072
ENSMUSP00000006669
ENSMUSP00000006669
ENSRNOP00000002072
ENSCAFP00000041833
ENSCAFP00000019265
ENSP00000376352
ENSPTRP00000065011
ENSMODP00000039449
ENSMODP00000011372
ENSMODP00000011266
ENSGALP00000021580
ENSTNIP00000020062
ENSDARP00000022009
ENSGALP00000015774
ENSMODP00000058442
ENSP00000005178
ENSPTRP00000033224
ENSCAFP00000003118
ENSRNOP00000012759
ENSMUSP00000019721
ENSDARP00000071589
ENSDARP00000042188
ENSTNIP00000004633
ENSTNIP00000006761
ENSTNIP00000020088
ENSDARP00000150746
ENSDARP00000012861
ENSGALP00000060241
ENSMODP00000015067
ENSRNOP00000005641
ENSMUSP00000041447
ENSCAFP00000024969
ENSPTRP00000015943
ENSP00000420927
ENSDARP00000138167
ENSDARP00000153715
ENSTNIP00000010986
ENSGALP00000026284
ENSMODP00000009219
ENSRNOP00000068033
ENSMUSP00000036604
ENSPTRP00000037297
ENSP00000498864
ENSCAFP00000019855
FBpp0111805
WBGene00022719.1
ENSCINP00000003914
