# Supplementary material for: Evolutionary Study of Protein Short Tandem Repeats in Protein Families
Source: Biomolecules. 2023 Jul 13;13(7):1116. doi: 10.3390/biom13071116 (PMC10377733; doi:10.3390/biom13071116)
Supplement: Supplementary file 1 [file biomolecules-13-01116-s001.zip › Suppl.FileS1.zip!/Suppl.File1/OG37/OG37_results.pdf]

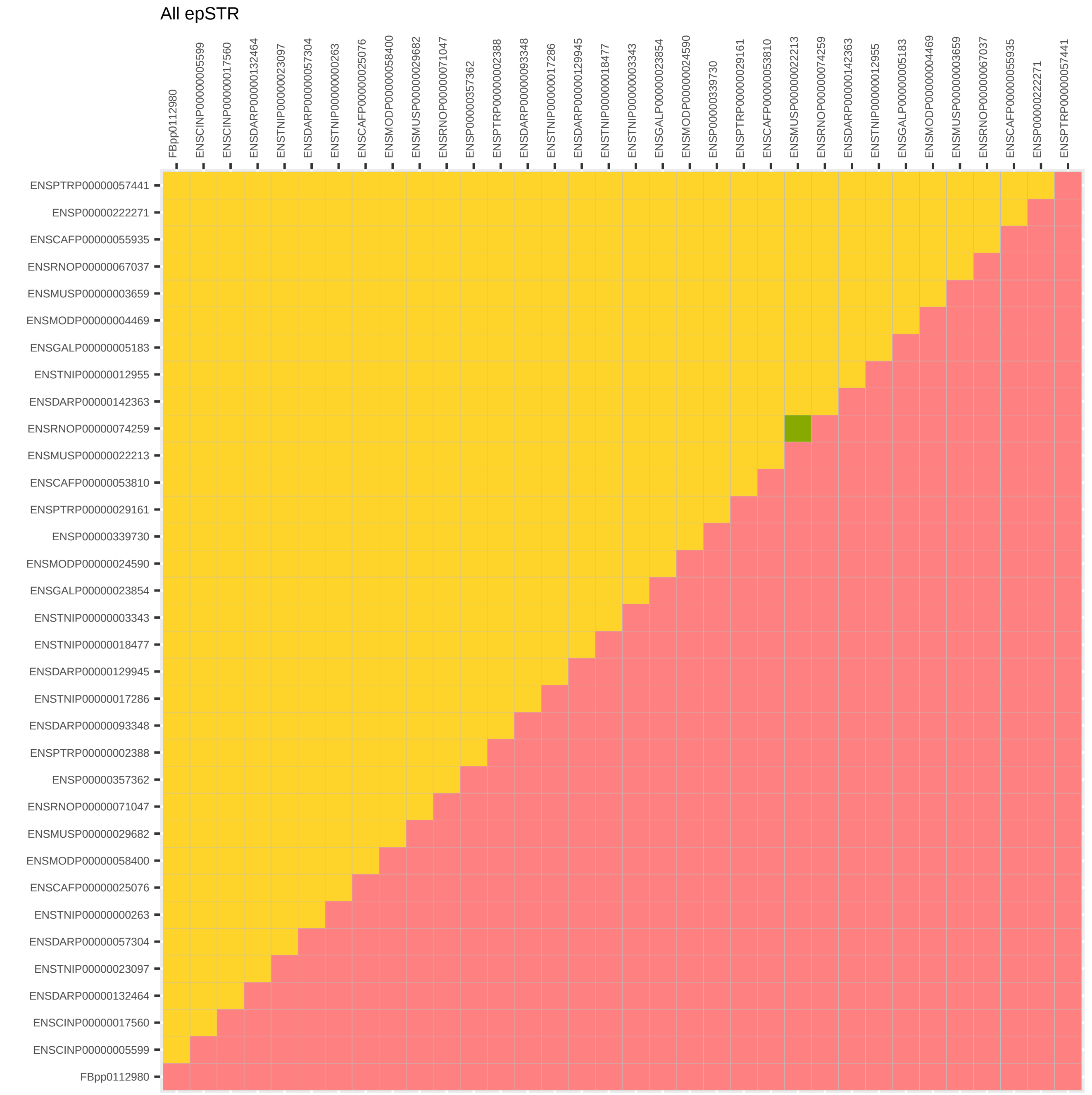
All epSTR
FBpp0112980
ENSCINP00000005599
ENSCINP00000017560
ENSDARP00000132464
ENSTNIP00000023097
ENSDARP00000057304
ENSTNIP00000000263
ENSCAFP00000025076
ENSMODP00000058400
ENSMUSP00000029682
ENSRNOP00000071047
ENSP00000357362
ENSPTRP00000002388
ENSDARP00000093348
ENSTNIP00000017286
ENSDARP00000129945
ENSTNIP00000018477
ENSTNIP00000003343
ENSGALP00000023854
ENSMODP00000024590
ENSP00000339730
ENSPTRP00000029161
ENSCAFP00000053810
ENSMUSP00000022213
ENSRNOP00000074259
ENSDARP00000142363
ENSTNIP00000012955
ENSGALP00000005183
ENSMODP00000004469
ENSMUSP00000003659
ENSRNOP00000067037
ENSCAFP00000055935
ENSP00000222271
ENSPTRP00000057441
