# Supplementary material for: Evolutionary Study of Protein Short Tandem Repeats in Protein Families
Source: Biomolecules. 2023 Jul 13;13(7):1116. doi: 10.3390/biom13071116 (PMC10377733; doi:10.3390/biom13071116)
Supplement: Supplementary file 1 [file biomolecules-13-01116-s001.zip › Suppl.FileS1.zip!/Suppl.File1/OG40/OG40_results.pdf]

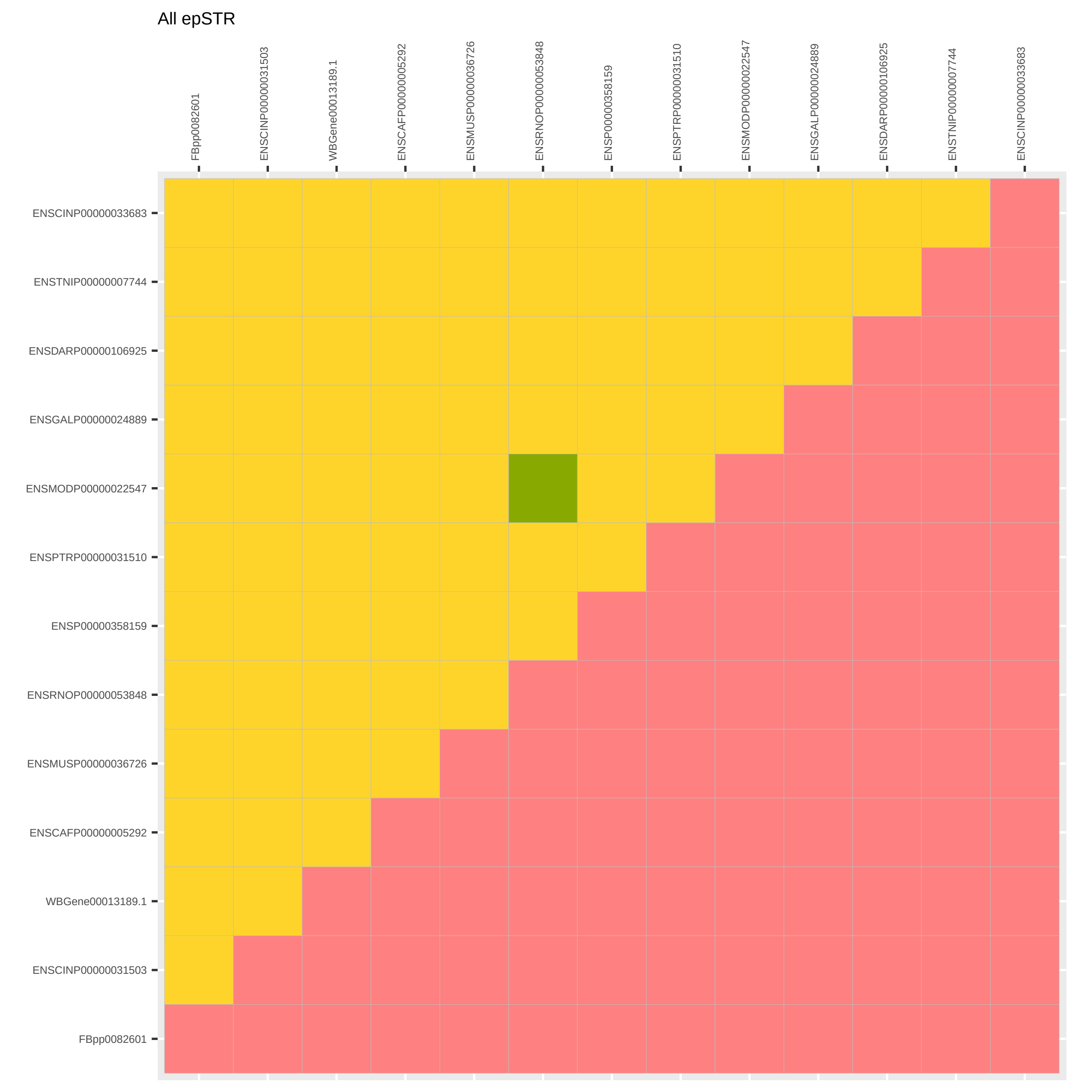
All epSTR
FBpp0082601
ENSCINP00000031503
WBGene00013189.1
ENSCAFP00000005292
ENSMUSP00000036726
ENSRNOP00000053848
ENSP00000358159
ENSPTRP00000031510
ENSMODP00000022547
ENSGALP00000024889
ENSDARP00000106925
ENSTNIP00000007744
ENSCINP00000033683
