# Supplementary material for: Evolutionary Study of Protein Short Tandem Repeats in Protein Families
Source: Biomolecules. 2023 Jul 13;13(7):1116. doi: 10.3390/biom13071116 (PMC10377733; doi:10.3390/biom13071116)
Supplement: Supplementary file 1 [file biomolecules-13-01116-s001.zip › Suppl.FileS1.zip!/Suppl.File1/OG54/OG54_results.pdf]

All epSTR

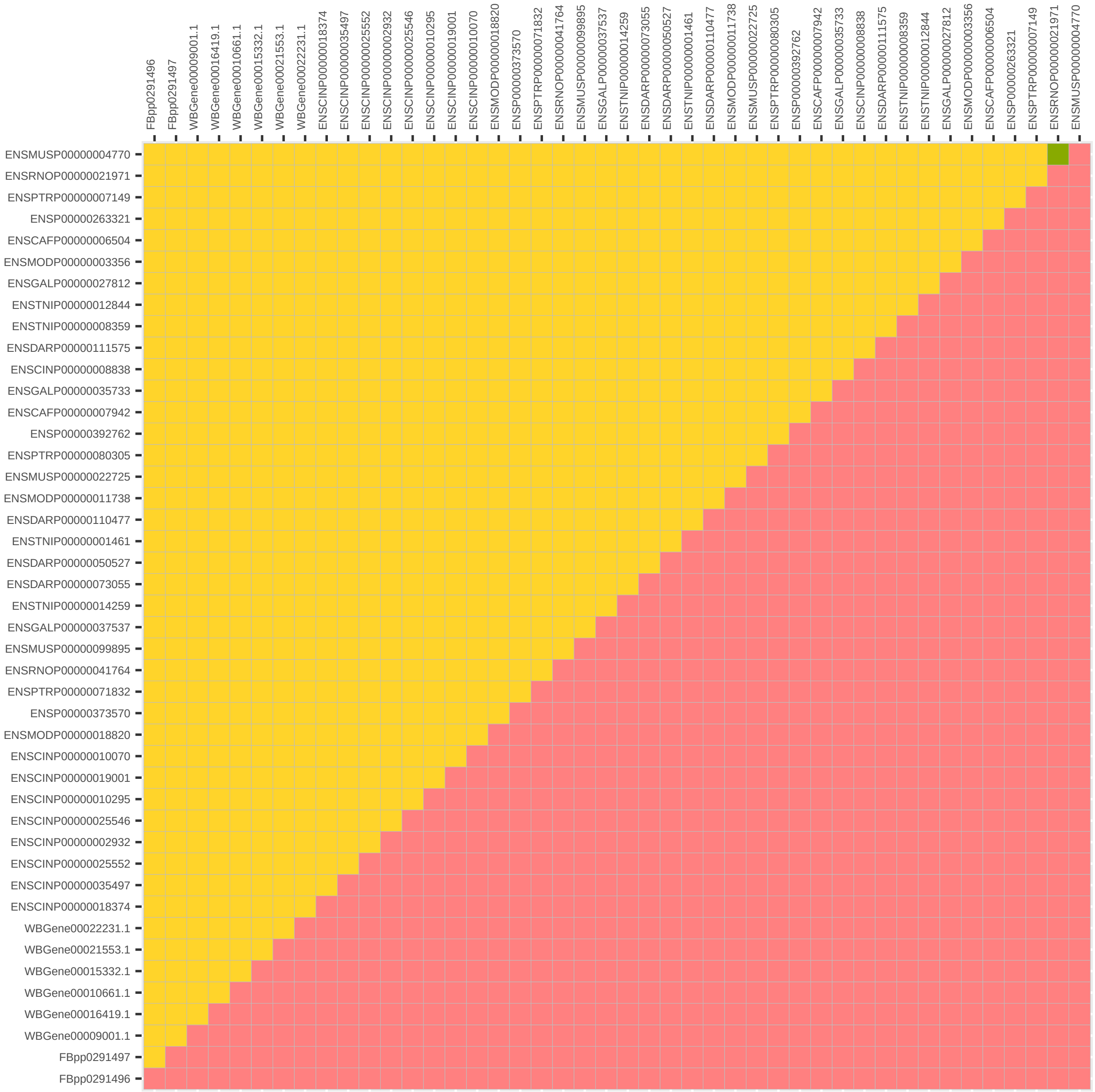
FBpp0291496
FBpp0291497
WBGene00009001.1
WBGene00016419.1
WBGene00010661.1
WBGene00015332.1
WBGene00021553.1
WBGene00022231.1
ENSCINP00000018374
ENSCINP00000035497
ENSCINP00000025552
ENSCINP00000002932
ENSCINP00000025546
ENSCINP00000010295
ENSCINP00000019001
ENSCINP00000010070
ENSMODP00000018820
ENSP00000373570
ENSPTRP00000071832
ENSRNOP00000041764
ENSMUSP00000099895
ENSGALP00000037537
ENSTNIP00000014259
ENSDARP00000073055
ENSDARP00000050527
ENSTNIP00000001461
ENSDARP00000110477
ENSMODP00000011738
ENSMUSP00000022725
ENSPTRP00000080305
ENSP00000392762
ENSCAFP00000007942
ENSGALP00000035733
ENSCINP00000008838
ENSDARP00000111575
ENSTNIP00000008359
ENSTNIP00000012844
ENSGALP00000027812
ENSMODP00000003356
ENSCAFP00000006504
ENSP00000263321
ENSPTRP00000007149
ENSRNOP00000021971
ENSMUSP00000004770
