# Supplementary material for: Evolutionary Study of Protein Short Tandem Repeats in Protein Families
Source: Biomolecules. 2023 Jul 13;13(7):1116. doi: 10.3390/biom13071116 (PMC10377733; doi:10.3390/biom13071116)
Supplement: Supplementary file 1 [file biomolecules-13-01116-s001.zip › Suppl.FileS1.zip!/Suppl.File1/OG56/OG56_results.pdf]

All epSTR

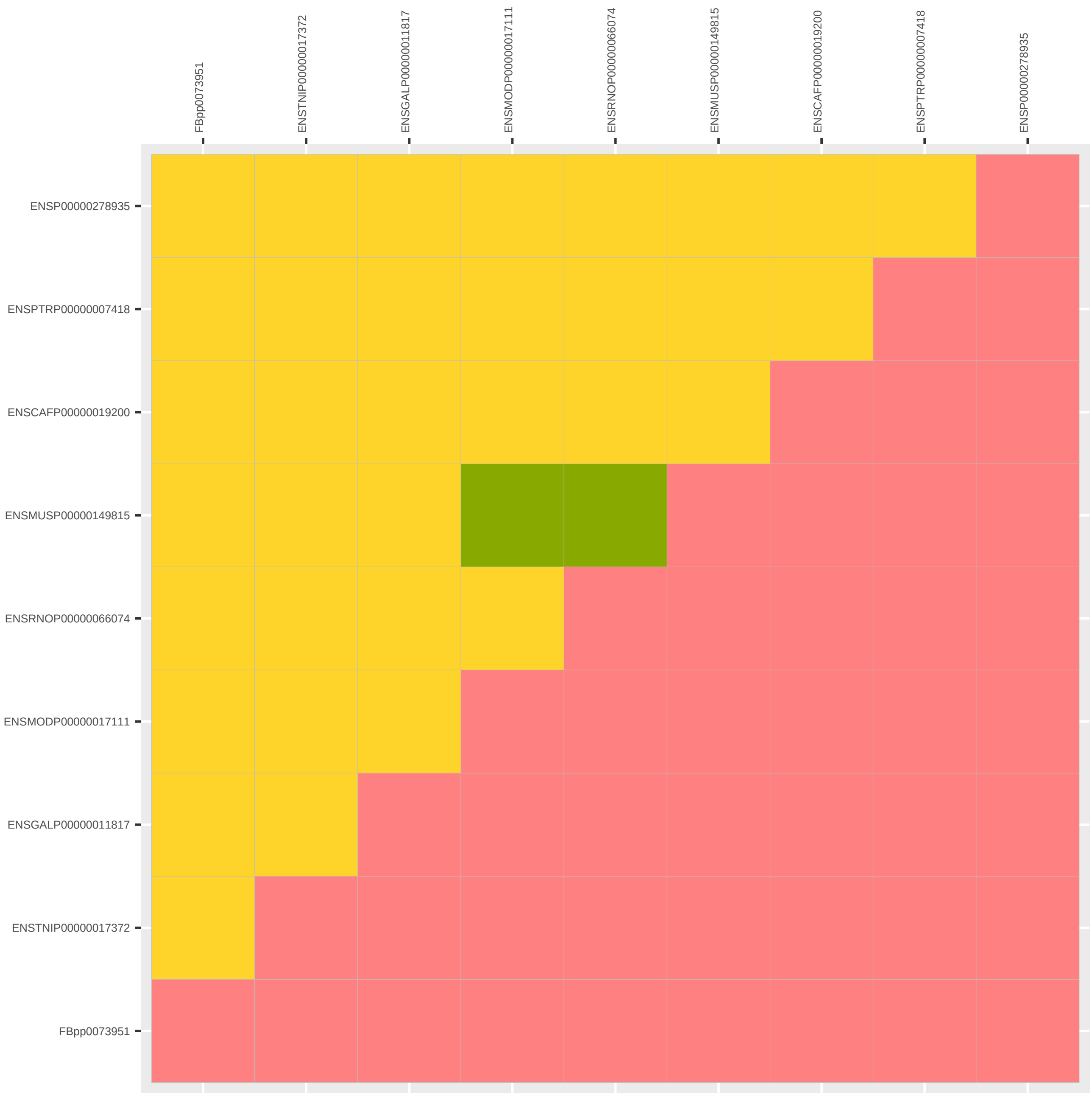
FBpp0073951
ENSTNIP00000017372
ENSGALP00000011817
ENSMODP00000017111
ENSRNOP00000066074
ENSMUSP00000149815
ENSCAFP00000019200
ENSPTRP00000007418
ENSP00000278935
ENSP00000278935
ENSPTRP00000007418
ENSCAFP00000019200
ENSMUSP00000149815
ENSRNOP00000066074
ENSMODP00000017111
ENSGALP00000011817
ENSTNIP00000017372
FBpp0073951
