# Supplementary material for: Evolutionary Study of Protein Short Tandem Repeats in Protein Families
Source: Biomolecules. 2023 Jul 13;13(7):1116. doi: 10.3390/biom13071116 (PMC10377733; doi:10.3390/biom13071116)
Supplement: Supplementary file 1 [file biomolecules-13-01116-s001.zip › Suppl.FileS1.zip!/Suppl.File1/OG59/OG59_results.pdf]

All epSTR

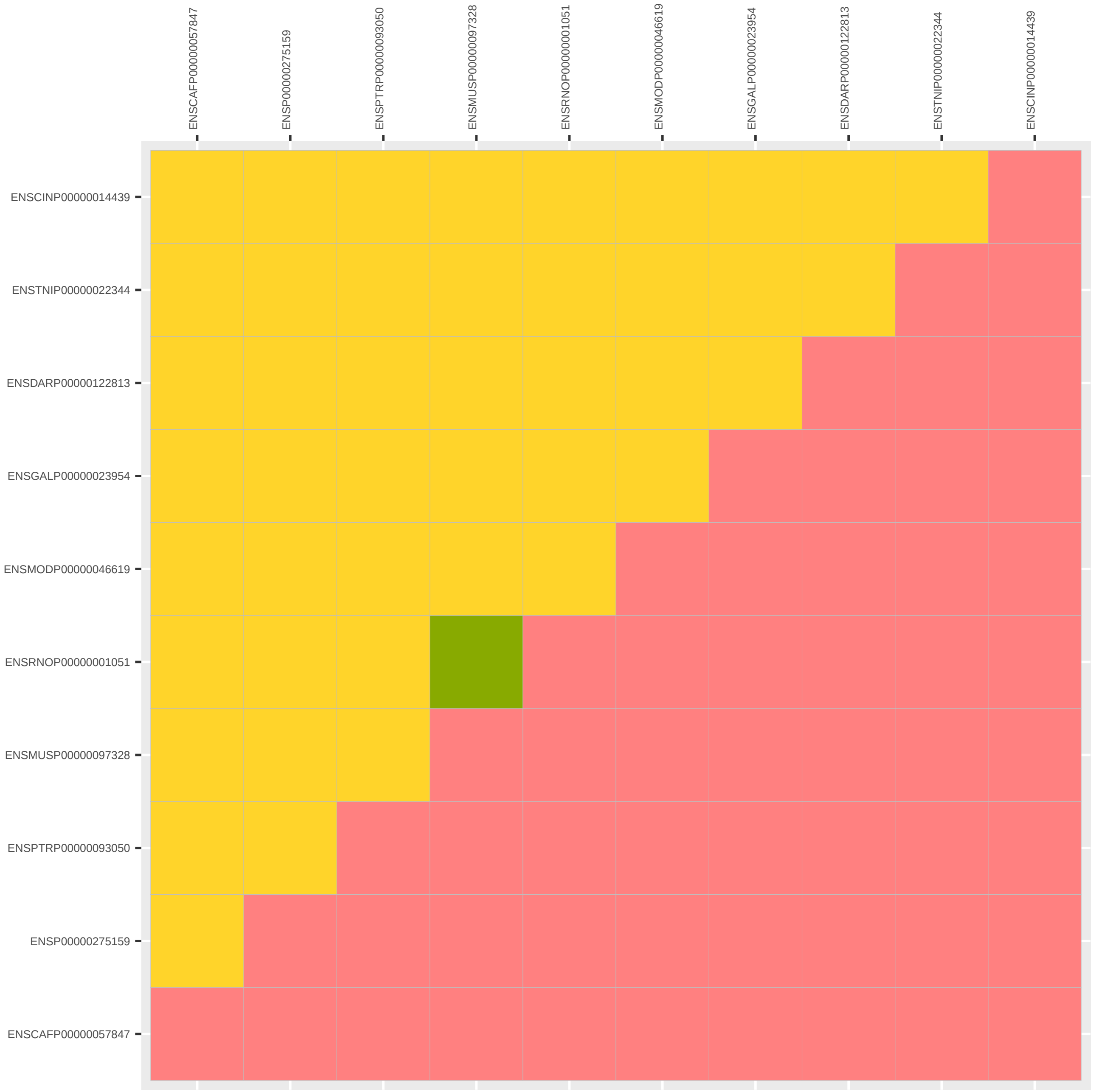
ENSCAFP00000057847
ENSP00000275159
ENSPTRP00000093050
ENSMUSP00000097328
ENSRNOP00000001051
ENSMODP00000046619
ENSGALP00000023954
ENSDARP00000122813
ENSTNIP00000022344
ENSCINP00000014439
