# Supplementary material for: Evolutionary Study of Protein Short Tandem Repeats in Protein Families
Source: Biomolecules. 2023 Jul 13;13(7):1116. doi: 10.3390/biom13071116 (PMC10377733; doi:10.3390/biom13071116)
Supplement: Supplementary file 1 [file biomolecules-13-01116-s001.zip › Suppl.FileS1.zip!/Suppl.File1/OG61/OG61_epSTRdistribution.pdf]

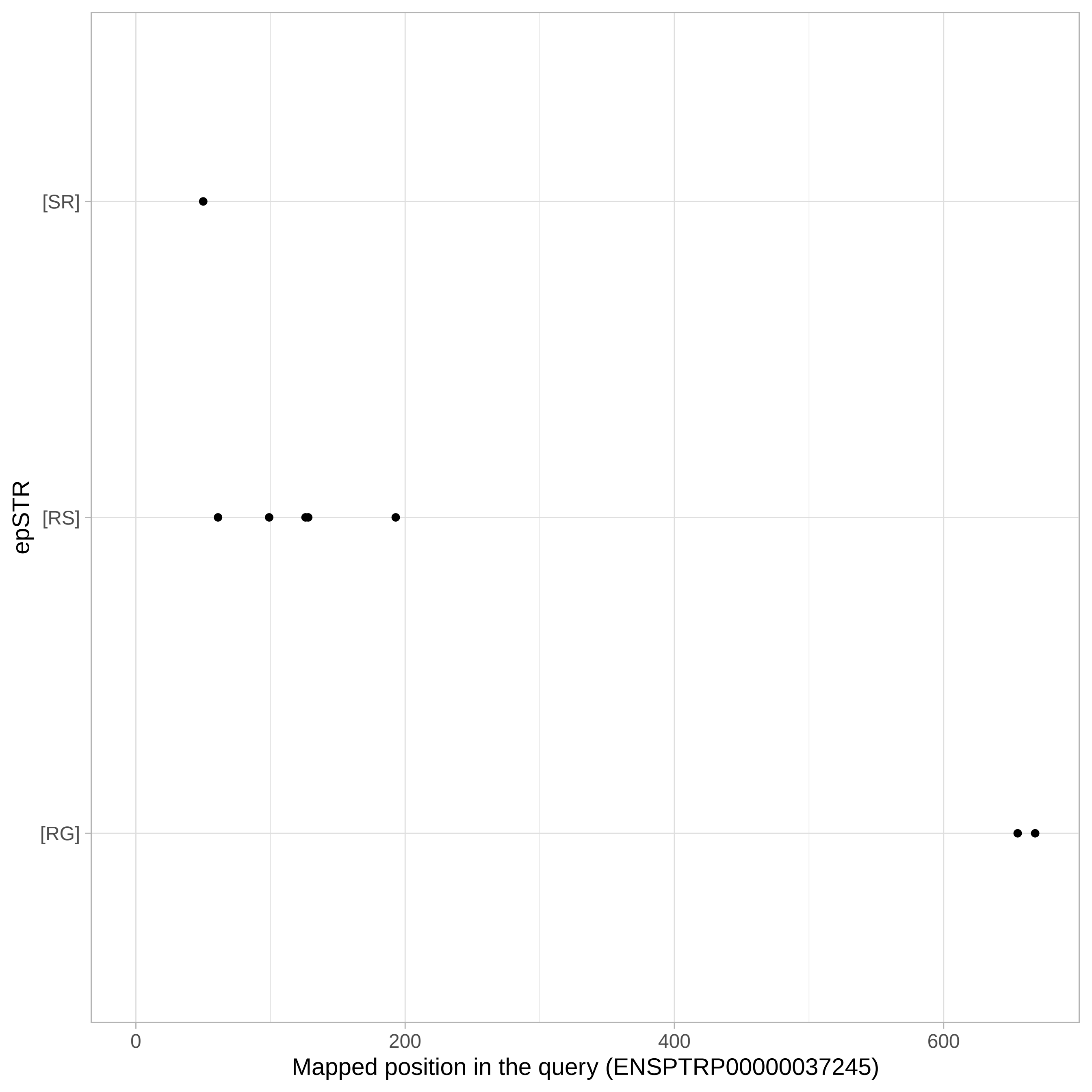
epSTR
[SR]
[RS]
[RG]
0
200
400
600
Mapped position in the query (ENSPTRP00000037245)
