# Supplementary material for: Evolutionary Study of Protein Short Tandem Repeats in Protein Families
Source: Biomolecules. 2023 Jul 13;13(7):1116. doi: 10.3390/biom13071116 (PMC10377733; doi:10.3390/biom13071116)
Supplement: Supplementary file 1 [file biomolecules-13-01116-s001.zip › Suppl.FileS1.zip!/Suppl.File1/OG61/OG61_results.pdf]

All epSTR

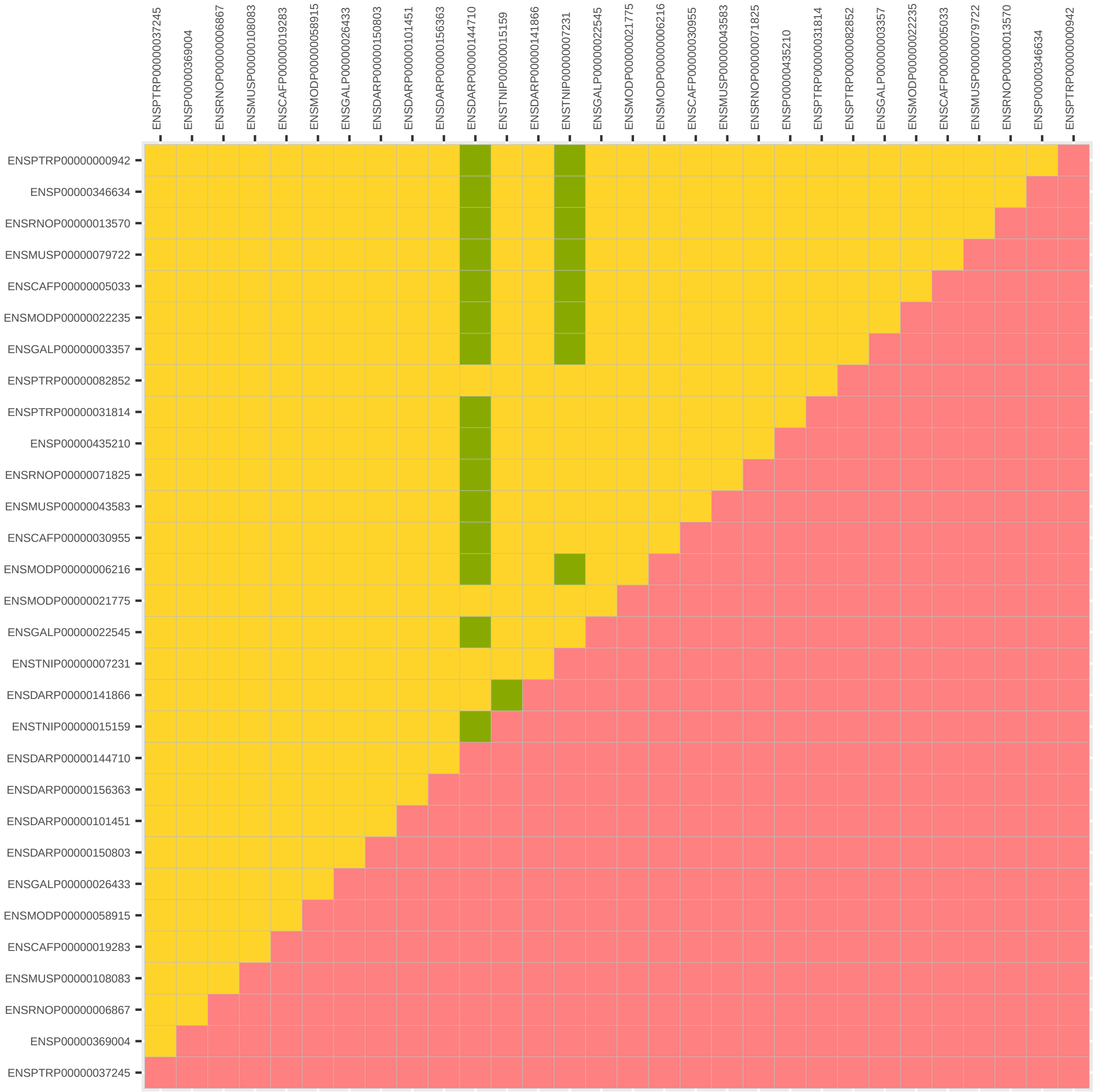
ENSPTRP00000037245
ENSP00000369004
ENSRNOP00000006867
ENSMUSP00000108083
ENSCAFP00000019283
ENSMODP00000058915
ENSGALP00000026433
ENSDARP00000150803
ENSDARP00000101451
ENSDARP00000156363
ENSDARP00000144710
ENSTNIP00000015159
ENSDARP00000141866
ENSTNIP00000007231
ENSGALP00000022545
ENSMODP00000021775
ENSMODP00000006216
ENSCAFP00000030955
ENSMUSP00000043583
ENSRNOP00000071825
ENSP00000435210
ENSPTRP00000031814
ENSPTRP00000082852
ENSGALP00000003357
ENSMODP00000022235
ENSCAFP00000005033
ENSMUSP00000079722
ENSRNOP00000013570
ENSP00000346634
ENSPTRP00000000942
