# Supplementary material for: Evolutionary Study of Protein Short Tandem Repeats in Protein Families
Source: Biomolecules. 2023 Jul 13;13(7):1116. doi: 10.3390/biom13071116 (PMC10377733; doi:10.3390/biom13071116)
Supplement: Supplementary file 1 [file biomolecules-13-01116-s001.zip › Suppl.FileS1.zip!/Suppl.File1/OG68/OG68_results.pdf]

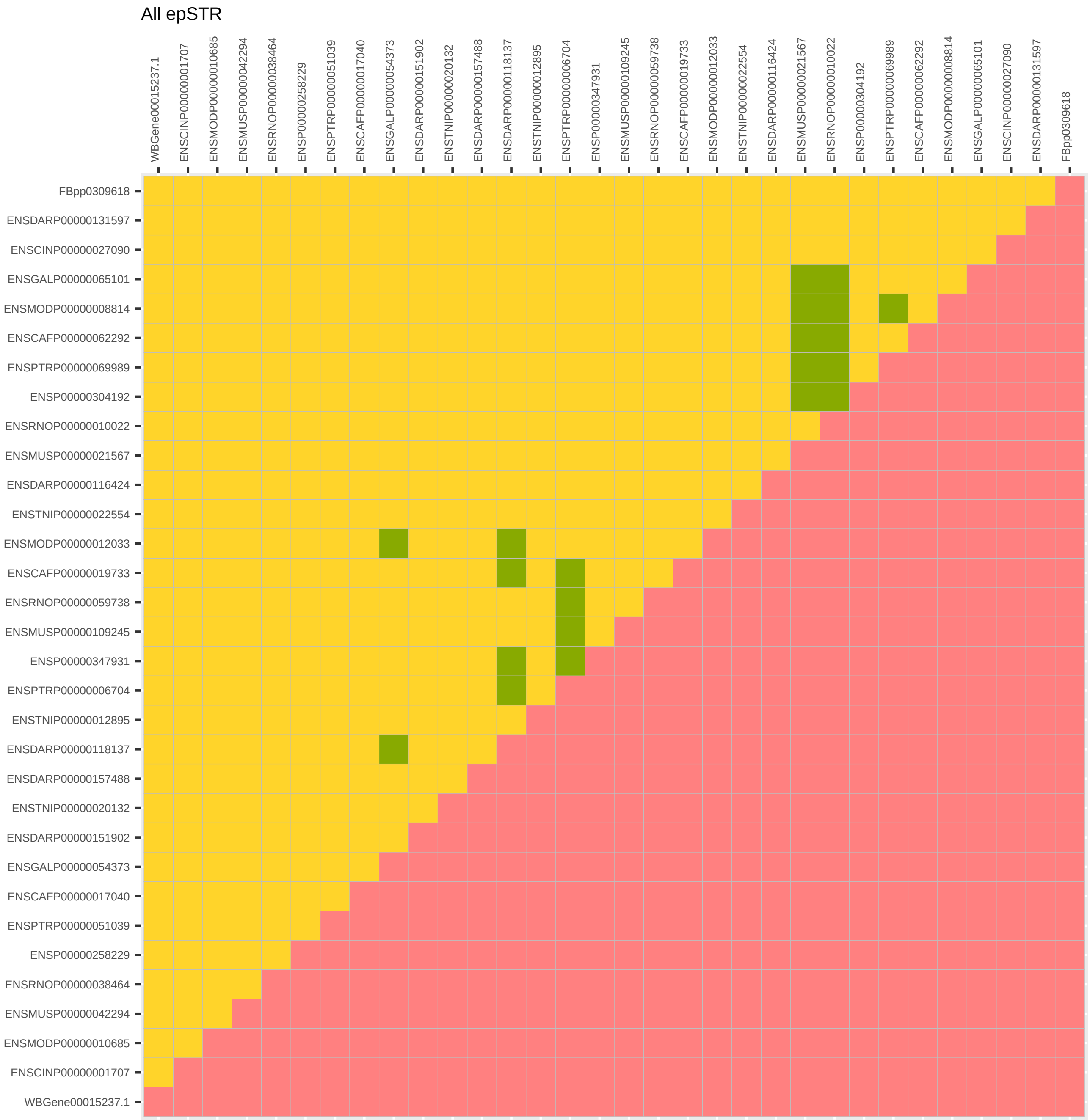
All epSTR
WBGene00015237.1
ENSCINP00000001707
ENSMODP00000010685
ENSMUSP00000042294
ENSRNOP00000038464
ENSP00000258229
ENSPTRP00000051039
ENSCAFP00000017040
ENSGALP00000054373
ENSDARP00000151902
ENSTNIP00000020132
ENSDARP00000157488
ENSDARP00000118137
ENSTNIP00000012895
ENSPTRP00000006704
ENSP00000347931
ENSMUSP00000109245
ENSRNOP00000059738
ENSCAFP00000019733
ENSMODP00000012033
ENSTNIP00000022554
ENSDARP00000116424
ENSMUSP00000021567
ENSRNOP00000010022
ENSP00000304192
ENSPTRP00000069989
ENSCAFP00000062292
ENSMODP00000008814
ENSGALP00000065101
ENSCINP00000027090
ENSDARP00000131597
FBpp0309618
