# Supplementary material for: Evolutionary Study of Protein Short Tandem Repeats in Protein Families
Source: Biomolecules. 2023 Jul 13;13(7):1116. doi: 10.3390/biom13071116 (PMC10377733; doi:10.3390/biom13071116)
Supplement: Supplementary file 1 [file biomolecules-13-01116-s001.zip › Suppl.FileS1.zip!/Suppl.File1/OG6/OG6_results.pdf]

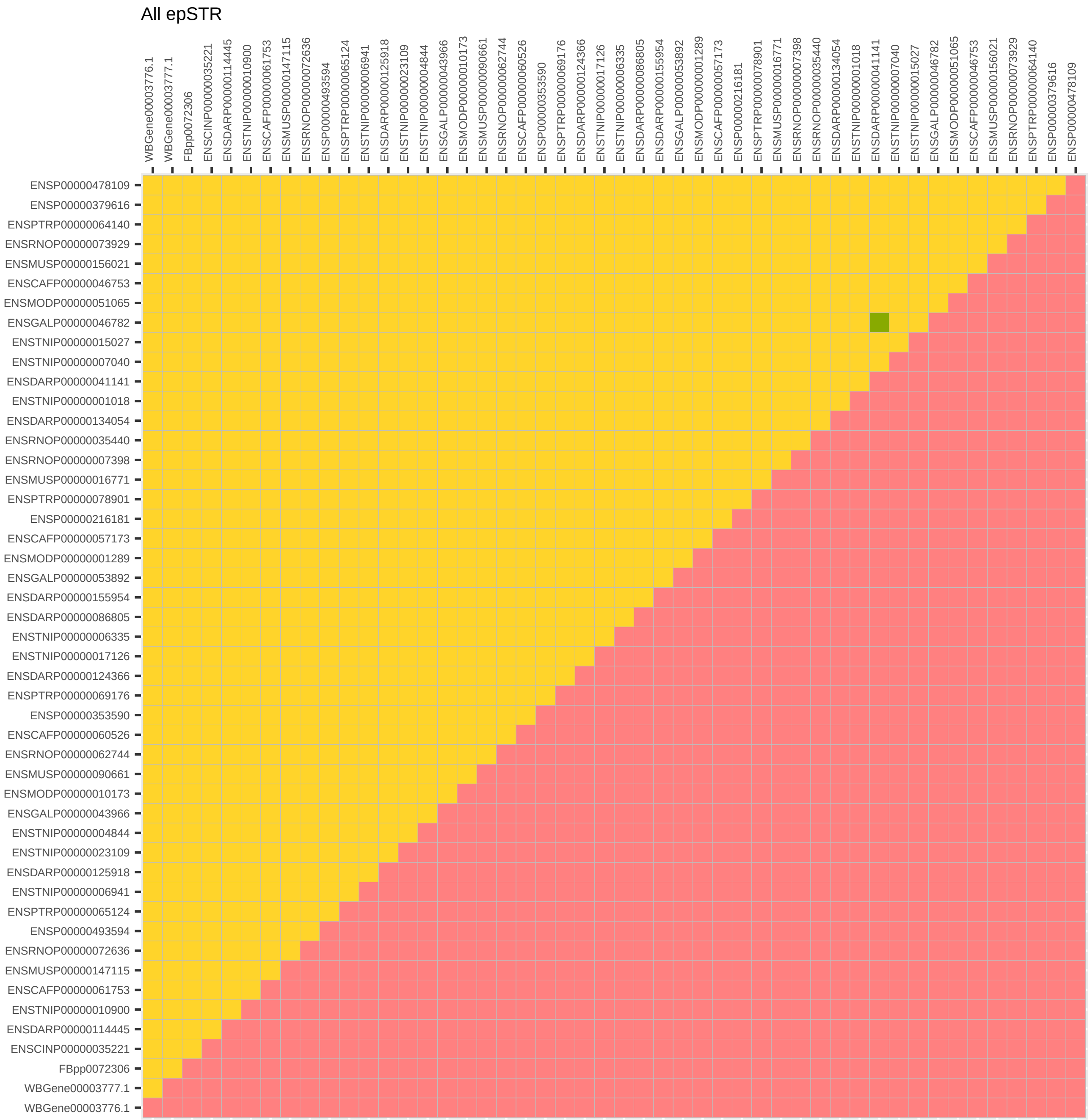
All epSTR
WBGene00003776.1
WBGene00003777.1
FBpp0072306
ENSCINP00000035221
ENSDARP00000114445
ENSTNIP00000010900
ENSCAFP00000061753
ENSMUSP00000147115
ENSRNOP00000072636
ENSP00000493594
ENSPTRP00000065124
ENSTNIP00000006941
ENSDARP00000125918
ENSTNIP00000023109
ENSTNIP00000004844
ENSGALP00000043966
ENSMODP00000010173
ENSMUSP00000090661
ENSRNOP00000062744
ENSCAFP00000060526
ENSP00000353590
ENSPTRP00000069176
ENSDARP00000124366
ENSTNIP00000017126
ENSTNIP00000006335
ENSDARP00000086805
ENSDARP00000155954
ENSGALP00000053892
ENSMODP00000001289
ENSCAFP00000057173
ENSP00000216181
ENSPTRP00000078901
ENSMUSP00000016771
ENSRNOP00000007398
ENSRNOP00000035440
ENSDARP00000134054
ENSTNIP00000001018
ENSDARP00000041141
ENSTNIP00000007040
ENSTNIP00000015027
ENSGALP00000046782
ENSMODP00000051065
ENSCAFP00000046753
ENSMUSP00000156021
ENSRNOP00000073929
ENSPTRP00000064140
ENSP00000379616
ENSP00000478109
