# Supplementary material for: Evolutionary Study of Protein Short Tandem Repeats in Protein Families
Source: Biomolecules. 2023 Jul 13;13(7):1116. doi: 10.3390/biom13071116 (PMC10377733; doi:10.3390/biom13071116)
Supplement: Supplementary file 1 [file biomolecules-13-01116-s001.zip › Suppl.FileS1.zip!/Suppl.File1/OG7/OG7_results.pdf]

All epSTR

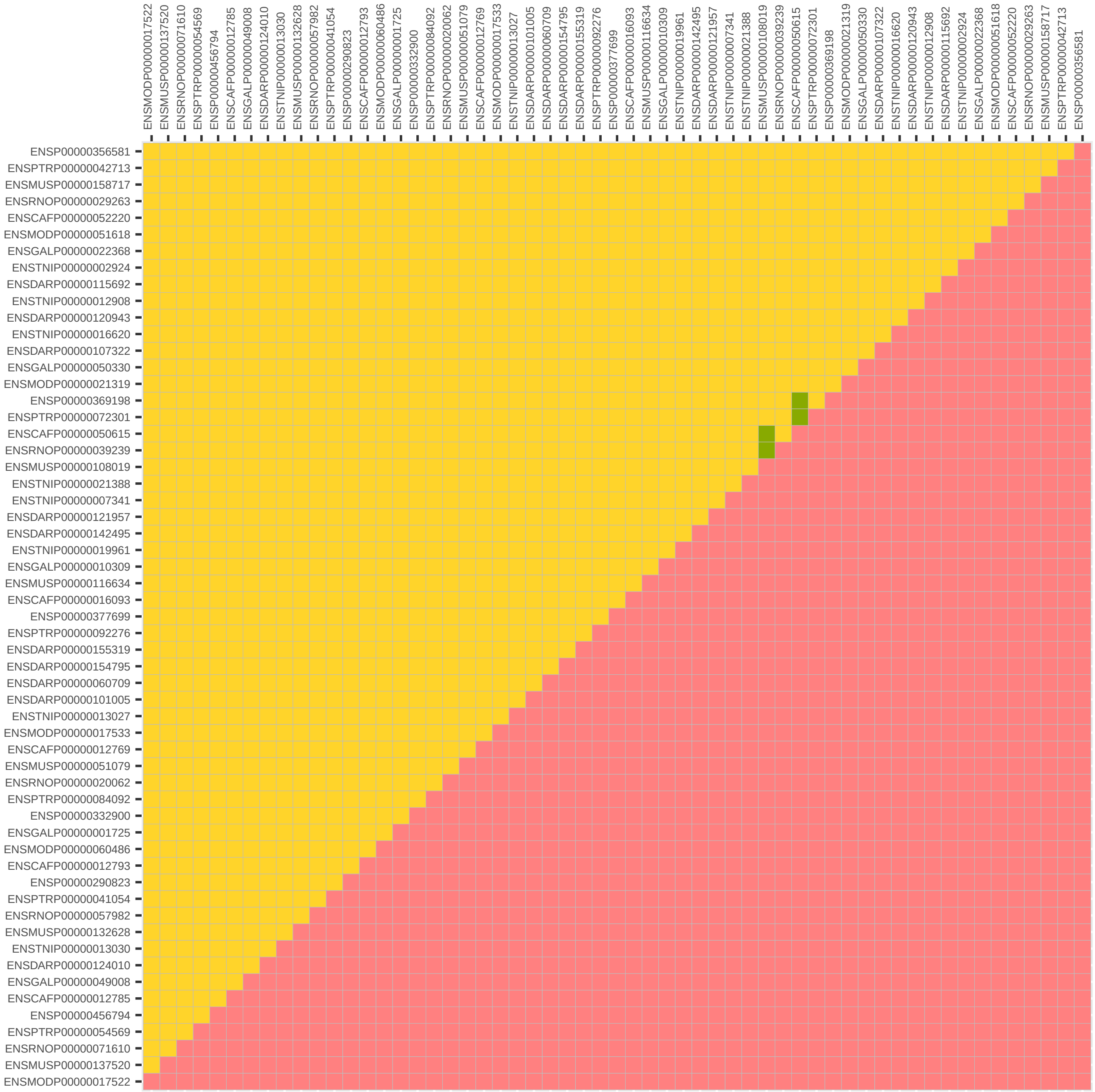
ENSMODP00000017522
ENSMUSP00000137520
ENSRNOP00000071610
ENSPTRP00000054569
ENSP00000456794
ENSCAFP00000012785
ENSGALP00000049008
ENSDARP00000124010
ENSTNIP00000013030
ENSMUSP00000132628
ENSRNOP00000057982
ENSPTRP00000041054
ENSP00000290823
ENSCAFP00000012793
ENSMODP00000060486
ENSGALP00000001725
ENSP00000332900
ENSPTRP00000084092
ENSRNOP00000020062
ENSMUSP00000051079
ENSCAFP00000012769
ENSMODP00000017533
ENSTNIP00000013027
ENSDARP00000101005
ENSDARP00000060709
ENSDARP00000154795
ENSDARP00000155319
ENSPTRP00000092276
ENSP00000377699
ENSCAFP00000016093
ENSMUSP00000116634
ENSGALP00000010309
ENSTNIP00000019961
ENSDARP00000142495
ENSDARP00000121957
ENSTNIP00000007341
ENSTNIP00000021388
ENSMUSP00000108019
ENSRNOP00000039239
ENSCAFP00000050615
ENSPTRP00000072301
ENSP00000369198
ENSMODP00000021319
ENSGALP00000050330
ENSDARP00000107322
ENSTNIP00000016620
ENSDARP00000120943
ENSTNIP00000012908
ENSDARP00000115692
ENSTNIP00000002924
ENSGALP00000022368
ENSMODP00000051618
ENSCAFP00000052220
ENSRNOP00000029263
ENSMUSP00000158717
ENSPTRP00000042713
ENSP00000356581
ENSP00000356581
ENSPTRP00000042713
ENSMUSP00000158717
ENSRNOP00000029263
ENSCAFP00000052220
ENSMODP00000051618
ENSGALP00000022368
ENSTNIP00000002924
ENSDARP00000115692
ENSTNIP00000012908
ENSDARP00000120943
ENSTNIP00000016620
ENSDARP00000107322
ENSGALP00000050330
ENSMODP00000021319
ENSP00000369198
ENSPTRP00000072301
ENSCAFP00000050615
ENSRNOP00000039239
ENSMUSP00000108019
ENSTNIP00000021388
ENSTNIP00000007341
ENSDARP00000121957
ENSDARP00000142495
ENSTNIP00000019961
ENSGALP00000010309
ENSMUSP00000116634
ENSCAFP00000016093
ENSP00000377699
ENSPTRP00000092276
ENSDARP00000155319
ENSDARP00000154795
ENSDARP00000060709
ENSDARP00000101005
ENSTNIP00000013027
ENSMODP00000017533
ENSCAFP00000012769
ENSMUSP00000051079
ENSRNOP00000020062
ENSPTRP00000084092
ENSP00000332900
ENSGALP00000001725
ENSMODP00000060486
ENSCAFP00000012793
ENSP00000290823
ENSPTRP00000041054
ENSRNOP00000057982
ENSMUSP00000132628
ENSTNIP00000013030
ENSDARP00000124010
ENSGALP00000049008
ENSCAFP00000012785
ENSP00000456794
ENSPTRP00000054569
ENSRNOP00000071610
ENSMUSP00000137520
ENSMODP00000017522
