# Supplementary material for: Evolutionary Study of Protein Short Tandem Repeats in Protein Families
Source: Biomolecules. 2023 Jul 13;13(7):1116. doi: 10.3390/biom13071116 (PMC10377733; doi:10.3390/biom13071116)
Supplement: Supplementary file 1 [file biomolecules-13-01116-s001.zip › Suppl.FileS1.zip!/Suppl.File1/OG9/OG9_results.pdf]

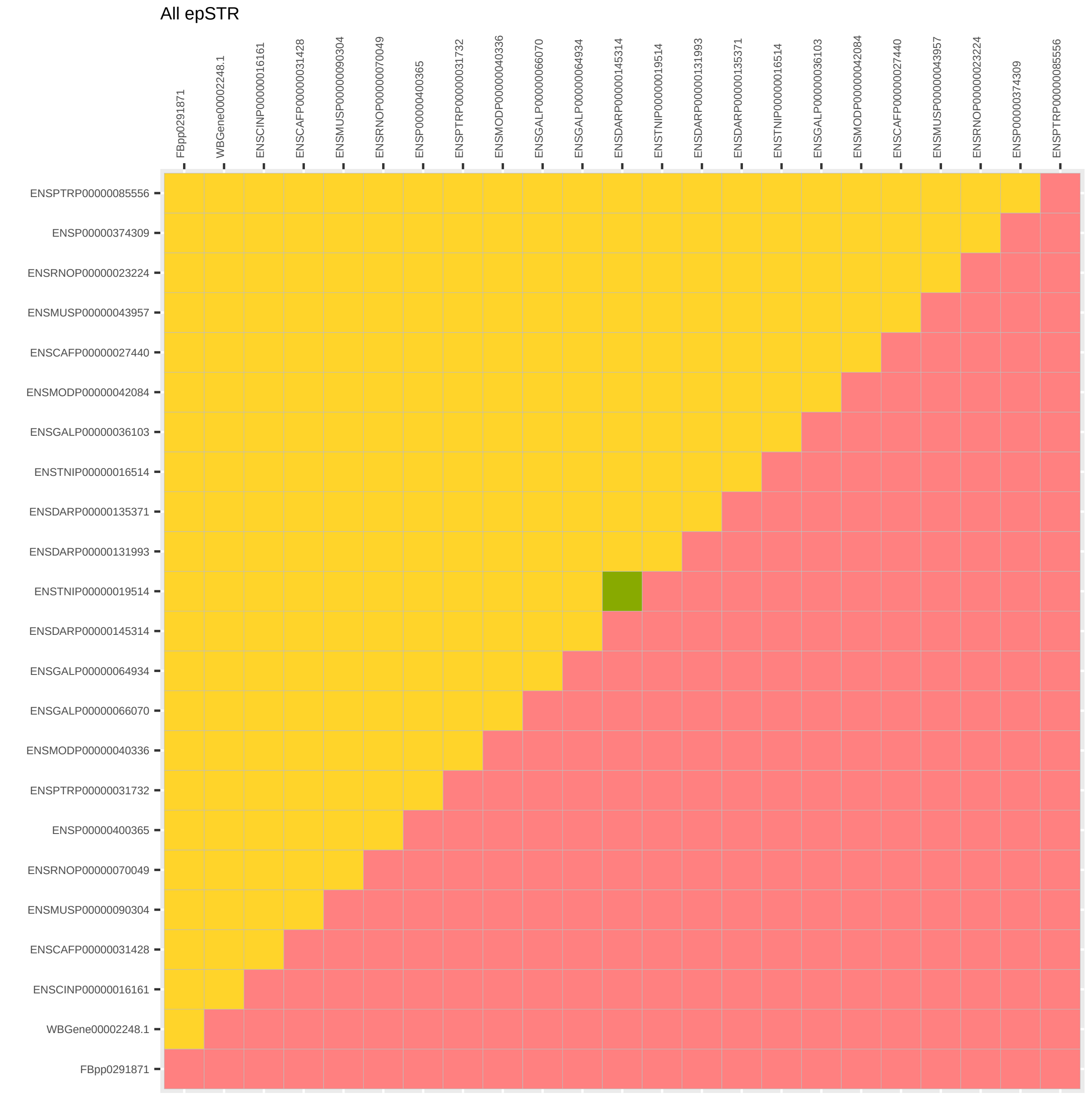
All epSTR
FBpp0291871
WBGene00002248.1
ENSCINP00000016161
ENSCAFP00000031428
ENSMUSP00000090304
ENSRNOP00000070049
ENSP00000400365
ENSPTRP00000031732
ENSMODP00000040336
ENSGALP00000066070
ENSGALP00000064934
ENSDARP00000145314
ENSTNIP00000019514
ENSDARP00000131993
ENSDARP00000135371
ENSTNIP00000016514
ENSGALP00000036103
ENSMODP00000042084
ENSCAFP00000027440
ENSMUSP00000043957
ENSRNOP00000023224
ENSP00000374309
ENSPTRP00000085556
